# Chronic, Low-Dose Methamphetamine Reveals Sexual Dimorphism of Memory Performance, Histopathology, and Gene Expression Affected by HIV-1 Tat Protein in a Transgenic Model of NeuroHIV

**DOI:** 10.3390/v17030361

**Published:** 2025-02-28

**Authors:** Indira S. Harahap-Carrillo, Dominic Fok, Frances Wong, Gabriel Malik, Ricky Maung, Xinru Qiu, Daniel Ojeda-Juárez, Victoria E. Thaney, Ana B. Sanchez, Adam Godzik, Amanda J. Roberts, Marcus Kaul

**Affiliations:** 1 Division of Biomedical Sciences, School of Medicine, University of California, Riverside (UCR), Riverside, CA 92521, USA; indira.harahapcarrillo@ucr.edu (I.S.H.-C.); dominic.fok@email.ucr.edu (D.F.); frances.wong@email.ucr.edu (F.W.); gabriel.malik@email.ucr.edu (G.M.); ricky.maung@ucr.edu (R.M.); xinru.qiu@ucr.edu (X.Q.); dojedajuarez@health.ucsd.edu (D.O.-J.); adam.godzik@ucr.edu (A.G.); 2 Neuroscience Graduate Program, College of Natural & Agricultural Sciences, University of California, Riverside (UCR), Riverside, CA 92521, USA; 3 Translational Methamphetamine AIDS Research Center (TMARC), Department of Psychiatry, University of California, San Diego (UCSD), San Diego, CA 92093, USA; absanmar@gmail.com; 4 Center for Infectious and Inflammatory Disease, Sanford Burnham Prebys Medical Discovery Institute (SBP), La Jolla, CA 92037, USA; victoriathaneysd@gmail.com; 5 The Scripps Research Institute (TSRI), Animal Models Core, La Jolla, CA 92037, USA; aroberts@scripps.edu

**Keywords:** HIV-1, Tat, methamphetamine, neurodegeneration, sexual dimorphism

## Abstract

Methamphetamine (METH) use is frequent among people with HIV (PWH) and appears to increase the risk of neuronal injury and neurocognitive impairment (NCI). This study explored in vivo the effects of a 12 week (long-term), low-dose METH regimen in a transgenic animal model of neuroHIV with inducible expression of HIV-1 transactivator of transcription (Tat). Seven months after transient Tat induction and five months after METH exposure ended, we detected behavioral changes in the Barnes maze (BM) spatial memory task in the Tat and METH groups but not the combined Tat + METH group. The novel object recognition (NOR) task revealed that Tat extinguished discrimination in female animals with and without METH, although METH alone slightly improved NOR. In contrast, in males, Tat, METH, and Tat + METH all compromised NOR. Neuropathological examination detected sex-dependent and brain region-specific changes of pre-synaptic terminals, neurites, and activation of astrocytes and microglia. RNA-sequencing and quantitative reverse transcription polymerase chain reaction indicated that METH and Tat significantly altered gene expression, including factors linked to Alzheimer’s disease-like NCI. In summary, chronic low-dose METH exerts long-term effects on behavioral function, neuropathology, and mRNA expression, and modulates the effects of Tat, suggesting sex-dependent and -independent mechanisms may converge in HIV brain injury and NCI.

## 1. Introduction

Despite the success of combined antiretroviral therapy (cART), up to 50% of people with HIV (PWH) continue to develop neurocognitive impairment (NCI), which clinically is referred to as HIV-associated neurocognitive disorders (HAND) [1,2,3]. The neuropathological features of HIV infection of the brain (neuroHIV) are often referred to as HIV encephalitis (HIVE) and include astrogliosis, microgliosis, and synaptic and dendritic loss [4,5,6]. Methamphetamine (METH) use is highly prevalent in PWH, and the interaction of HIV and psychostimulant drug appears to worsen HAND severity [3,7]. METH alone or in combination with HIV-1 causes oxidative stress, excitotoxicity, and mitochondrial dysfunction [8]. PWH who use METH show increased neuronal injury, NCI, and viral load [9,10]. Overall, METH has typically been found to increase the risk of neuronal dysfunction, independently of and as a comorbidity of HIV-1 infection.

Although, METH is a commonly abused schedule II drug, it is also clinically administered at low concentrations to patients as a treatment for attention deficit hyperactivity disorder (ADHD) [11]. In addition, recent studies have suggested that METH can ameliorate neuronal damage during traumatic brain injury (TBI) and mild Alzheimer’s disease (AD) [12,13]. Moreover, an epidemiological study of patterns of METH use reported that many METH users consuming limited dosages apparently remain neurocognitively functional despite long-term exposure for up to 21 years [14]. Together, these observations suggest that the dosage of METH may play a critical role in determining whether METH exposure leads to a therapeutic or detrimental effect.

The viral genome of HIV is made up of genes encoding structural and regulatory viral proteins, such as gp160 (gp120 and gp41) and Tat, Vpr, and Nef, respectively [15]. Previous studies have shown that individual viral proteins of HIV-1 can cause direct or indirect injury resulting in neuronal damage [16,17,18]. In a study of our lab in a transgenic neuroHIV/HAND animal model expressing HIV-1 envelope protein gp120 in the brain, a METH binge regimen showed an amelioration in cortical dendritic damage, while the overall effect on behavior and neurons was deleterious [19]. This finding supports the notion that the dosage is pivotal in determining the extent of ameliorating versus detrimental outcomes of exposure to METH. Based on the observations of long-term, limited dose use patterns [14], we explore in the present study the effects of a long-term (12 week), low-dose exposure to METH in a mouse model of neuroHIV that expresses the inducible regulatory HIV Tat protein in the brain [20]. The inducible Tat–transgenic mice (iTat) express the viral protein upon treatment with doxycycline, resulting in neuropathology and behavioral impairment [20,21]. Expression of Tat alone can induce key features of neuroHIV, including activation of neuroglia, dysregulation of presynaptic protein, increased cytokine release, and disruption of amyloid beta (Aβ) degradation [15,20,22,23]. In the present study we subjected iTat mice at 4 months of age to a 12-week METH regimen. During week 4, the mice received doxycycline (Dox) for transient induction of Tat expression. Four months after METH exposure, at 11–12 months of age, all iTat mice underwent behavioral testing, including novel object recognition (NO) and the Barnes maze test (BM). Tat compromised performance in the NO and BM paradigms, but the combination of METH and Tat resulted unexpectedly in a virtually normal performance in the probe trial of the BM. However, the NO paradigm revealed a sexual dimorphism in that METH alone only compromised males, whereas females, in contrast to males, displayed no discrimination between familiar and novel object after exposure to Tat with or without METH. Histopathological and gene expression analysis, including RNA-sequencing, further reveal sex-dependent effects of both Tat and METH.

Altogether, our findings suggest that a chronic low dose of METH has long-term effects on behavioral performance, neuropathology and mRNA expression and can modulate some detrimental effects of Tat. This scenario also suggests that a sex-dependent interplay of neuronal stimulation and neuroinflammatory pathways may converge in brain injury and promote the development of NCI/HAND.

## 2. Materials and Methods

### 2.1. Animal Model and Drug Treatment

The transgenic iTat mouse expresses the HIV-1 Tat protein under control of a Dox-inducible glial fibrillary acidic protein (GFAP) promoter [20]. The bigenic mouse model employs two transgenes (GFAP-rtTA^+^/pTRE-Tat^+^) enabling the inducible expression of viral Tat. In the Tet-ON-system, Dox is a tetracycline (Tet) analog, that allows the binding of the reverse tetracycline-controlled transactivator (rtTA) to bind the operator allowing for transcription of the target gene [24]. The iTat mice have been shown to display induced Tat expression for more than 2 weeks after Dox injections [21]. The iTat mouse is a clinically relevant model of HAND, as Tat has been shown to be present in the central nervous system (CNS) and cerebrospinal fluid (CSF) of PLWH [25]. In addition, the inducible nature of Tat in this animal model allows for a non-constitutive expression, avoiding expression of the protein during development [20].

An age and sex matched cohort of iTat mice was employed for this study. At four months of age, animals in the METH exposure groups, started receiving injections with 5 days of an escalating dosage of METH in week 1 (1 × s.c./day starting at 0.5 mg/kg, increasing step wise to 2.5 mg/kg, sterile-filtered solution of METH ((+)-Methamphetamine hydrochloride, M-8750, Sigma-Aldrich, St. Louis, WA, USA). Animals not receiving METH were given sterile saline (vehicle) in the same volume (100 µL/25 g body weight). The long-term, low-dose METH regimen (12 weeks via s.c. 1×/day, 5 days/week) continued for the rest of 11 weeks at 2.5 mg/kg, Mon-Fri. This METH intake schedule (weekend days excluded), is representative of a chronic use pattern observed in humans, who can ingest the drug orally, transnasally, or parenterally [14].

**Figure 1:**
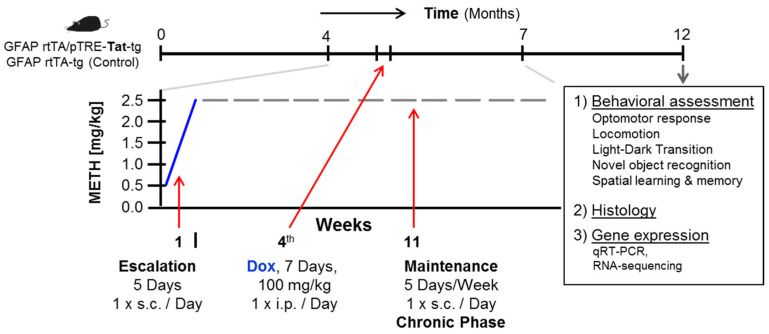
Schematic of overall experimental protocol. Mouse cohort consisted of two genotypes, GFAP-rtTA^+^/pTRE-Tat^+^ (40 mice) and GFAP-rtTA^+^/pTRE-Tat^−^ (43 mice) with 35 females and 48 males, 83 mice total. The 12-week METH regimen entailed the following steps: Week 1, starting at 0.5 mg/kg s.c., 1 × day, step-wise increase by 0.5 mg/kg with each injection over 5 days (Mon–Fri), followed by 11 weeks 1 × 2.5 mg/kg/day (Mon–Fri = 20+ days per month). Controls received vehicle (saline). All animals were treated with Doxycyclin (Dox).

All animal groups received Dox (Clontech Laboratories Inc., Mountain View, CA, USA, catalog #631311) during week 4 of the regimen (100 mg/kg for 7 days via i.p. 1×/day,) while METH/Saline injections continued (Figure 1). Hence, the four experimental groups were: **1.** ‘Ctl’—mice only expressing one transgene (GFAP-rtTA^+^), Tat-negative vehicle treated animal served as control, **2.** ‘Tat’—mice expressing both transgenes (GFAP-rtTA^+^/pTRE-Tat^+^), Tat-expressing vehicle treated animal, **3.** ‘METH’—mice expressing one transgene (GFAP-rtTA^+^), Tat-negative METH treated animals, and **4.** ‘Tat + METH’—mice expressing both transgenes (GFAP-rtTA^+^/pTRE-Tat^+^), Tat-expressing METH-treated animals.

All experimental procedures and protocols involving animals were performed in accordance with National Institute of Health Guide for the Care and Use of Laboratory Animals and were approved by the Institutional Animal Care and Use Committees (IACUC) of the University of California, Riverside, the Sanford Burnham Prebys Medical Discovery Institute, and The Scripps Research Institute.

### 2.2. Behavioral Testing

At 11 to 12 months of age, the animals of all experimental groups were sent for behavioral testing at The Scripps Research Institute’s (TSRI) Animal Models Core Facility. Animals included Ctl (male: 10, female: 9), Tat (male: 12, female: 7), METH (male: 11, female: 13), and Tat + METH (male: 15, female: 6). Behavioral testing was performed as previously described [26,27,28]. Briefly, mice were housed in standard plastic cages on a reversed 12 h light/dark cycle, with food and water available ad libitum. The behavioral test battery was designed to examine cognitive abilities, general activity, anxiety-like behavior, and visual capacity. The order of testing was as followed: light/dark transfer (LDT), locomotor activity (LM), optomotor (OM), novel object recognition (NOR), and Barnes maze (BM).

All procedures were approved by TSRI’s IACUC and met the guidelines of the National Institute of Health detailed in the *Guide for the Care and Use of Laboratory Animals*. Behavioral testing occurred between 9:00 AM and 12:00 PM (active phase) with 5–7 days between tests.

#### 2.2.1. Locomotor Activity Test

Locomotor activity was measured in polycarbonate cages (42 × 22 × 20 cm) placed into frames (25.5 × 47 cm) mounted with two levels of photocell beams at 2 and 7 cm above the bottom of the cage (San Diego Instruments, San Diego, CA, USA). These two sets of beams allowed for the recording of both horizontal (locomotion) and vertical (rearing) behavior. A thin layer of bedding material was applied to the bottom of the cage. Mice were tested for 120 min and data were collected in 5 min intervals.

#### 2.2.2. Optomotor Vision Test

The optomotor allows for assessment of visual ability and consists of a stationary elevated platform surrounded by a drum with black and white striped walls. Each mouse was placed on the platform to habituate for 1 min, then the drum rotated at 2 rpm in one direction for 1 min, stopped for 30 s, and rotated in the other direction for 1 min. The number of head tracks (15-degree movements at speed of drum) was recorded. Blind mice do not track the moving stripes.

#### 2.2.3. Light/Dark Transfer Test

The apparatus is a rectangular box made of Plexiglas divided by a partition into two environments. One compartment (14.5 × 27 × 26.5 cm) is dark (8–16 lux) and the other compartment (28.5 × 27 × 26.5 cm) is highly illuminated (400–600 lux) by a 60 W light source located above it. The compartments are connected by an opening (7.5 × 7.5 cm) located at floor level in the center of the partition. The time spent in the light compartment was used as a predictor of anxiety-like behavior, i.e., a greater amount of time in the light compartment was indicative of decreased anxiety-like behavior. Mice were placed in the dark compartment to start the 5 min test.

#### 2.2.4. Barnes Maze Test

The Barnes maze consists of an opaque Plexiglas disc 75 cm in diameter elevated 58 cm above the floor by a tripod. Twenty holes, 5 cm in diameter, are located 5 cm from the perimeter, and a black Plexiglas escape box (19 × 8 × 7 cm) is placed under one of the holes. Distinct spatial cues are located all around the maze and are kept constant throughout the study.

On the first day of testing, a training session was performed, which consisted of placing the mouse in the escape box and leaving it there for one minute. One minute later, the first session was started whereby the mouse was placed in the middle of the maze in a 10 cm high cone-shaped silver start chamber. After 10 s, the start chamber was removed, a buzzer (80 dB) and a light (400 lux) were turned on, and the mouse was set free to explore the maze. Sessions ended when the mouse entered the escape tunnel or after 3 min elapsed. When the mouse entered the escape tunnel, the buzzer was turned off and the mouse was allowed to remain in the dark for one minute. If the mouse did not enter the tunnel by itself, it was gently put in the escape box for one minute. The tunnel was always located underneath the same hole (stable within the spatial environment), which was randomly determined for each mouse. Mice were tested once a day for 4 days for the acquisition portion of the study.

A probe test was performed on the day following the final acquisition trial. For this test, the escape tunnel was removed and the mouse was allowed to freely explore the maze for 3 min. The time spent in each quadrant was determined and the percent time spent in the target quadrant (the one originally containing the escape box) was compared with the average percent time in the other three quadrants. This is a direct test of spatial memory as there are no local cues to be used in the mouse’s behavioral decision.

Each acquisition session was videotaped and scored by an experimenter blind to the genotype of the mouse. Measures recorded included the latency to escape the maze and the number of errors made per session. Errors were defined as nose pokes and head deflections over any hole that did not have the tunnel beneath it. Data were analyzed using Noldus EthoVision software (XT version 11.5; Noldus Information Technology, Leesburg, VA, USA) to determine time spent in each quadrant of the maze and to assess activity.

#### 2.2.5. Novel Object Recognition Test

Mice were tested with two identical objects placed in the field for 5 min. After three such trials (each separated by 1 min in a holding cage), one of the familiar objects was changed for a novel object. Habituation to the objects across the familiarization trials (decreased contacts) was an initial measure of learning, and renewed interest (increased contacts) in the new object indicated successful object memory.

### 2.3. Immunohistology

Mouse brains were harvested after the conclusion of the behavioral testing and immunostaining of sagittal was performed as previously published [19,26,29]. Briefly, for the purposes of the immunohistological studies, 8 animals from each group were collected (4 males and 4 females). To collect brain tissue, animals were terminally anesthetized with Isoflurane and transcardially perfused with 0.9% sterile saline. Brain tissues were collected after cervical transection and fixed with 4% paraformaldehyde in PBS (pH 7.4) for 48 h at 4 °C. For immunofluorescence (IF) staining, 40 µm thick floating sagittal brain sections were stained with antibodies against synaptophysin (mouse anti-synaptophysin, 1:50; Dako, Carpinteria, CA, USA, catalog #M7315) and MAP2 (mouse anti-microtubule-associated protein 2, 1:200; Sigma, catalog #M4403) for neuronal markers, Iba1 (rabbit anti-ionized calcium-binding adaptor molecule 1, 1:125; Wako, Osaka, Japan, catalog #01919241) and GFAP (rabbit anti-glial fibrillary acidic protein, 1:250; Dako, catalog #Z0334) for microglial and astrocytic markers, respectively. Secondary antibodies used were Alexa Fluor-488 goat anti-rabbit (1:200; Molecular Probes/Life Technologies, catalog #A11034) and Rhodamine Red goat anti-mouse (1:200; Jackson ImmunoResearch, West Grove, PA, USA, catalog #NC9046882) in 5% goat serum (PBS-T). Nuclear DNA was labeled with Hoechst 33342 (1:150; Sigma, catalog #B2261).

### 2.4. Imaging and Analysis

IF-stained tissues were imaged using a Zeiss 200 M fluorescence deconvolution microscope [19,26,27,29]. Capture and analysis of images was performed using SlideBook software (version 6; Intelligent Imaging Innovations, Inc., Denver, CO, USA). For each animal, three sagittal sections (slices were ~300 µm apart from each other) were stained, and each section was imaged in four non-overlapping fields within the target area, layer three (LIII) of the cortex and the cornu ammonis 1 (CA1) of the hippocampus. Two additional sections were used for staining controls: secondary antibody-only control and irrelevant IgG control for primary antibody. For synaptophysin (SYP), tissue sections were imaged at 40× (40×/0.75 Plan-NEOFLUAR, Zeiss, Oberkochen, Germany). The neuropil percentage occupied by SYP^+^ presynaptic terminals was determined by threshold segmentation for LIII of the cortex and CA1 of the hippocampus. MAP2 stained tissue sections were also imaged at 40×; MAP2^+^ neuropil percentage were determined in the same area and by the same method previously described [19,26,27,29]. GFAP stained tissue sections were imaged at 10× (10×/0.50 FLUAR, Zeiss, Oberkochen, Germany) and fluorescence in the total cortex and CA1 of the hippocampus were quantitatively analyzed. For Iba1, tissue sections were imaged at 10×, and Iba1^+^ microglia were counted in the CA1 of the hippocampus and LIII of the cortex.

### 2.5. Isolation of mRNA and Quantitative Reverse-Transcription Polymerase Chain Reaction (qRT-PCR)

Specific brain regions for RNA analysis were obtained by dissection during tissue harvest. Tissues of collected brain regions were quickly snap frozen using liquid nitrogen. Total RNA was isolated from frozen brain regions using the Qiagen RNeasy Lipid Tissue Midi Kit (Qiagen, Valencia, CA, USA). Of the total RNA isolated from each brain region’s tissue, an aliquot of 500 ng was used to produce cDNA through reverse transcription employing SuperScript II reverse transcriptase (Invitrogen, Life Technologies, Carlsbad, CA, USA). The cDNA was used to run a qRT-PCR using Power PCR SYBR Green Master Mix and the QuantStudio™ 6 Flex Real-Time PCR system. Each amplification reaction comprised 10 µL Power SYBR Green, 0.5 µL of each specific primer (forward and reverse from a 20 µM stock), and 9 µL of cDNA (25 ng). PCR amplification was completed using the following thermal conditions: 10 min–95 °C, 40 cycles of 15 sec–95 °C, and 1 min–60 °C. A set of primers was created for each gene of interest and *Gapdh* was used as a house keeping gene control in all qRT-PCR experiments for normalization of the data. The study followed MIQE guidelines [30]. For primer design, gene sequences were retrieved from the GenBank database (https://www.ncbi.nlm.nih.gov/, accessed on 20 February 2020). Using the gene sequences found, primers were designed for the experiment using Primer3 (version 2.5, Primer3.org) and Primer-BLAST software (https://www.ncbi.nlm.nih.gov/tools/primer-blast, accessed on 20 February 2020) and validated using serial dilutions of samples containing the target mRNA [26,27,29]. Table 1 lists the sequences of all primer sequences used.

### 2.6. RNA-Seq and Differential Gene Expression Analysis

RNA collected from the hippocampus was first bioanalyzed using an Agilent 2100 Biosystem/Tapestation in the University of California Riverside (UCR) Genomics Core. Samples with RIN > 7.0 (*n* = 3 males, 3 females per group) were sent to the University of California San Diego (UCSD) IGM Genomics Center (IGM), for RNA-sequencing (RNA-Seq) using rRNA depletion, PE100 paired-end reading and a sequencing depth of 25 M per sample. RNA libraries for RNA-seq were prepared by the UCSD (IGM): Illumina Total RNA Prep, Ribodepletion (following supplier’s instruction: enzymatic rRNA removal, ligation-based adapter, and index addition). For RNA-seq, the UCSD IGM Genomics Center utilized an Illumina NovaSeq X Plus; purchased with funding from a National Institutes of Health SIG grant (#S10 OD026929).

FASTQ files were then processed through the reference cDNA sequences for Mus musculus genome (GRCm39) which were downloaded from the Ensembl database (Release 110). Following the retrieval of this file, we utilized the Kallisto software (Version 0.50.0, Kallisto Software GmbH, Höxter, Germany) to generate an index that facilitates rapid transcript quantification [31]. This index was created by employing Kallisto’s index function on the downloaded cDNA FASTA file. For quantification, we executed the Kallisto quant command. The output includes estimates of transcript abundances, making it amenable to downstream analyses like differential gene expression studies. Read counts/TPM (transcripts per million) were converted into log2Fc using the DESeq2 script. Annotation was done on Galaxy (https://usegalaxy.eu/?tool_id=deseq2, accessed on 26 November 2023) using the Mus musculus genome reference [32]. Visualization of the differentially expressed genes employed a multitude of tools/scripts/software packages, including EnhancedVolcano, ShinyGO 0.77 (for GO Enrichment), and Ingenuity Pathway Analysis (IPA) for pathway and networks analysis. RNA-Seq data discussed in this paper have been deposited in NCBI’s Gene Expression Omnibus and are accessible through GEO Series accession number GSE255016 (https://www.ncbi.nlm.nih.gov/geo/query/acc.cgi?acc=GSE255016, accessed on 2 April 2024).

### 2.7. Statistical Analysis

Results from immunofluorescence staining and RNA expression analysis are presented as scatter plot with bar reflecting individual data points and mean with SEM. RNA expression data points from qRT-PCR analysis are averaged from two replicates of the same sample. Analysis and generation of graphs employed GraphPad Prism (Version 8, GraphPad Software, Inc., Boston, MA, USA). Comparison of more than two experimental groups used a one-way ANOVA, followed by Fisher’s PLSD post hoc test. Statistical significance was set as * *p* ≤ 0.05, ** *p* ≤ 0.01, *** *p* ≤ 0.001, **** *p* ≤ 0.0001.

This is the first study with this long-term METH regimen in the iTat mouse model. The histopathological analysis used here was the first time performed for this transgenic mouse model. Therefore, we did not specifically calculate a priori samples sizes for power but estimated the number of animals based on iTat-induced pathology seen in studies by others [20]. We also considered the behavioral study by another group and our previous experience with other transgenic mice [21,27,28,29,33]. That approach intended to assure that a sufficient number of animals is available for analysis of brain tissues after behavioral testing. Four animals per sex and experimental group were sufficient in previous studies to detect neuropathological differences between four experimental groups [26,27,28,29]. All iTat and Tat-negative control mice were from one founder line and heterozygous for their respective transgenes. Following the earlier studies by others [20,21,33], we decided to use Dox injection for all animals for the same amount of time in order to minimize variability of Tat induction.

## 3. Results

### 3.1. Behavioral Changes Due to Tat Expression and METH Treatment

**Figure 2:**
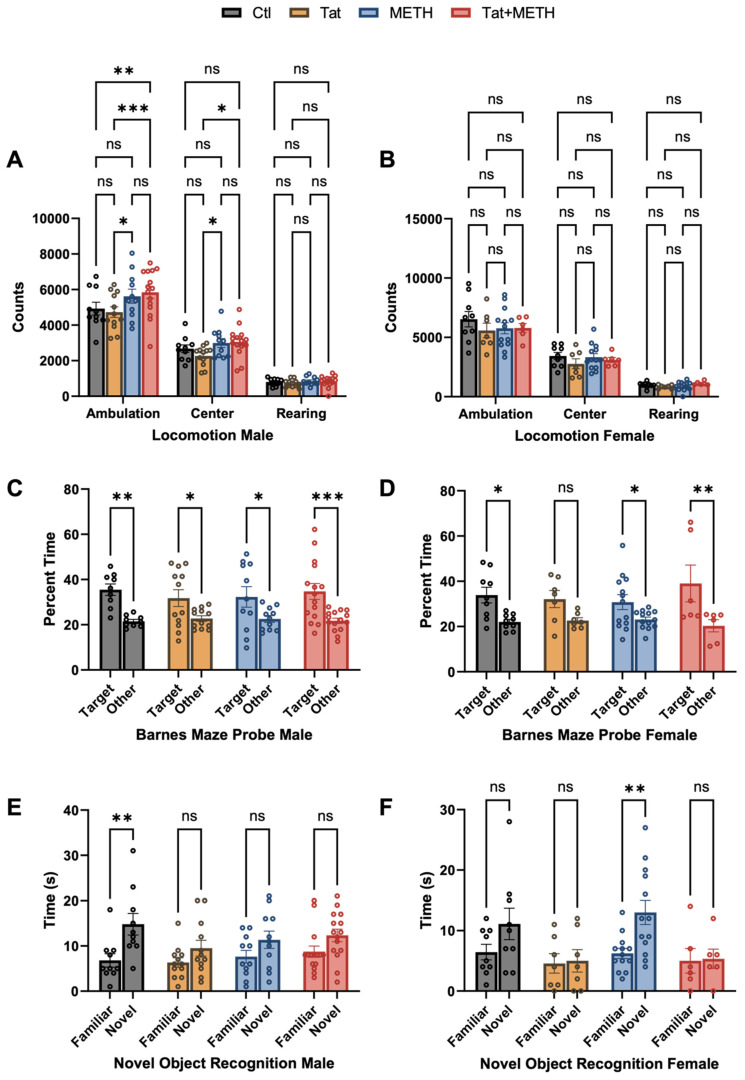
Learning and spatial memory impairment is affected by Tat expression and METH treatment. A cohort of 83 mice was subjected to behavioral assessment, *n* = Ctl (male: 10, female: 9), Tat (male: 12, female: 7), METH (male: 11, female: 13), and Tat + METH (male: 15, female: 6). (**A**,**B**) LM testing shows that female animals’ locomotor activity remained unaffected by Tat and METH or combination of both agents. In male animals, exposure to METH increased locomotor activity. (**C**) BM test was performed to assess spatial learning and memory in mice. In male animals, Tat expression and METH exposure both reduced spatial memory performance. However, Tat and METH in combination ameliorated deficit in spatial memory, resulting in performance being indistinguishable from control. (**D**) In female animals, only those with Tat expression alone had reduced spatial memory performance. (**E**,**F**) NOR test was performed to examine short term memory. Sex-dependent deficits in short term memory were observed. (**E**) Tat expression and METH exposure alone and in combination equally diminished novel object recognition in male animals. (**F**) In female animals, only METH exposed animals displayed novel object recognition reaching a significance level of *p* ≤ 0.01 while the Ctl group showed a trend (Familiar vs. Novel Ctl *p*-value = 0.0708). Values are mean ± SEM, * *p* ≤ 0.05, ** *p* ≤ 0.01, *** *p* ≤ 0.001, **** *p* ≤ 0.0001; ANOVA and Fisher’s PLSD post hoc test.

To investigate whether or not METH and Tat exposure following our experimental protocol caused behavioral deficits, the animals underwent a battery of behavioral tests as described in Materials and Methods and details of the statistical analysis are presented in Supplementary Table S1. The locomotor test (LM) showed the animals’ movement capability and activity (Figure 2A,B). Ambulation and center activity counts in male animals were slightly but significantly affected by the previous exposure to METH. Males of both METH and Tat + METH groups showed increased counts for ambulation and center activity compared to Tat group males. In addition, Tat + METH males had increased ambulation compared to control (Figure 2A). Rearing was unaffected by Tat or METH or the combination in animals of both sexes.

Optomotor responses (OM) showed head tracking in all groups indicating intact vision, with decreased numbers of head tracks in METH and Tat + METH animals (Figure S1A).

The measurement of time in light and light/dark transitions (LDT) showed no significant differences between experimental groups (Figure S1B,C).

The Barnes maze (BM) results showed sex-dependent effects of METH and Tat protein. Spatial memory of male mice was only slightly diminished (lesser significance for difference between target and other quadrants) by either METH or Tat alone but virtually unaffected in the Tat + METH group (Figure 2C,D). However, Tat reduced spatial memory in female mice (no significance for difference between target and other quadrants) while METH alone had no significant effect. Unexpectedly, METH exposure prevented the deficit associated with Tat expression and resulted in better performance of spatial memory in females (Figure 2C,D).

In the novel object recognition (NOR) test, we observed changes related to genotype and sex. NOR revealed that only male control animals (Ctl), showed significant discrimination between novel and familiar objects (Figure 2E). In male mice expression of Tat or METH exposure alone or combined significantly reduced the ability to distinguish between familiar and novel objects. On the other hand, in female mice, METH treatment improved recognition memory, and resulted in significant discrimination of novel and familiar objects. In contrast, Tat completely obliterated the distinction between the objects (Figure 2F). Moreover, METH treatment did not improve recognition memory of female or male animals with Tat protein (Figure 2E,F), indicating that the expression of Tat is the driving factor in the loss of ability in discrimination between novel and familiar objects.

In summary, Tat compromised spatial and recognition memory, as seen in the BM and NOR paradigms gradually more in females indicating a sex-dependent effect. METH exposure alone diminished NOR only in male mice; however, METH modulated and ameliorated the compromising effects of Tat on spatial memory in both sexes.

### 3.2. Neuronal Injury Associated with Tat Expression and METH Treatment

**Figure 3:**
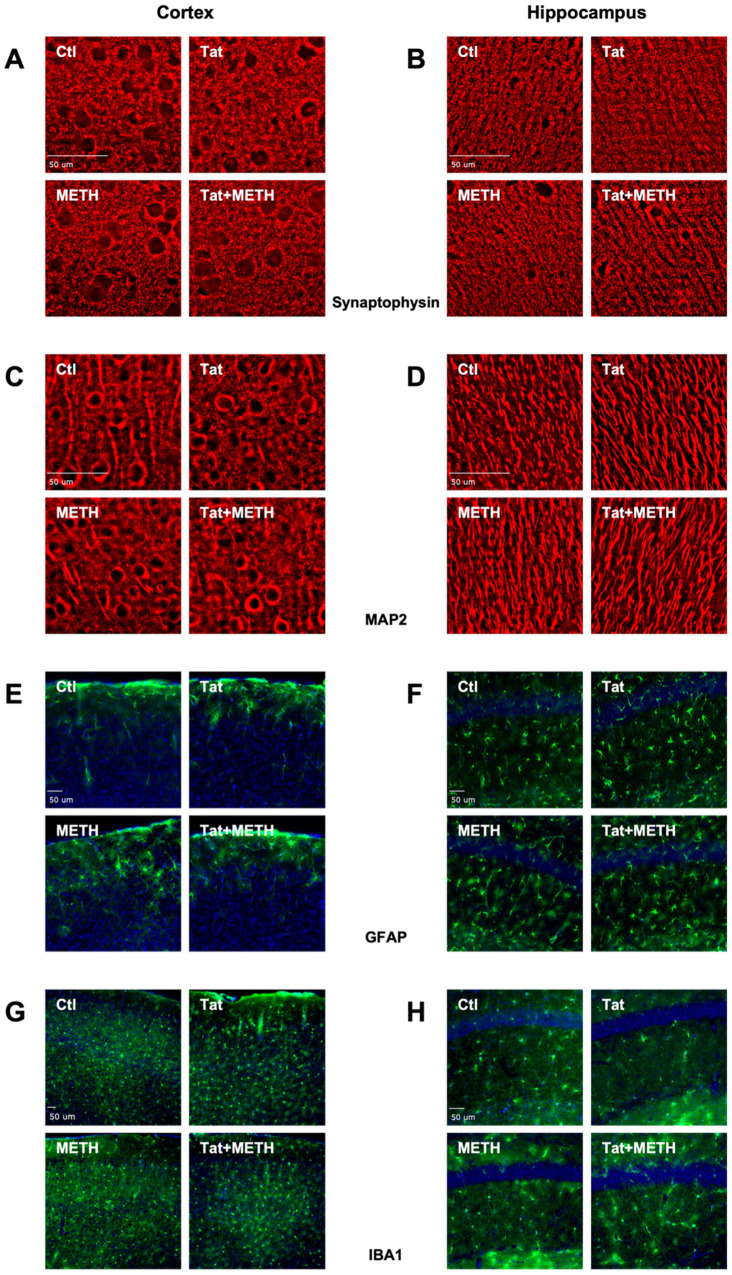
Immunofluorescence staining of cellular markers in cortex and hippocampus. Images of sagittal brain tissue sections stained for synaptophysin in (**A**) LIII of cortex and (**B**) CA1 of hippocampus. Images of tissue stained for MAP2 in (**C**) LIII of cortex and (**D**) CA1 of hippocampus. Images of tissue stained for GFAP in (**E**) total cortex and (**F**) CA1 of hippocampus. Images of tissue stained for Iba1 in (**G**) total cortex and (**H**) CA1 of hippocampus. Representative images taken from male animals are shown. Scale bar = 50 μm.

**Figure 4:**
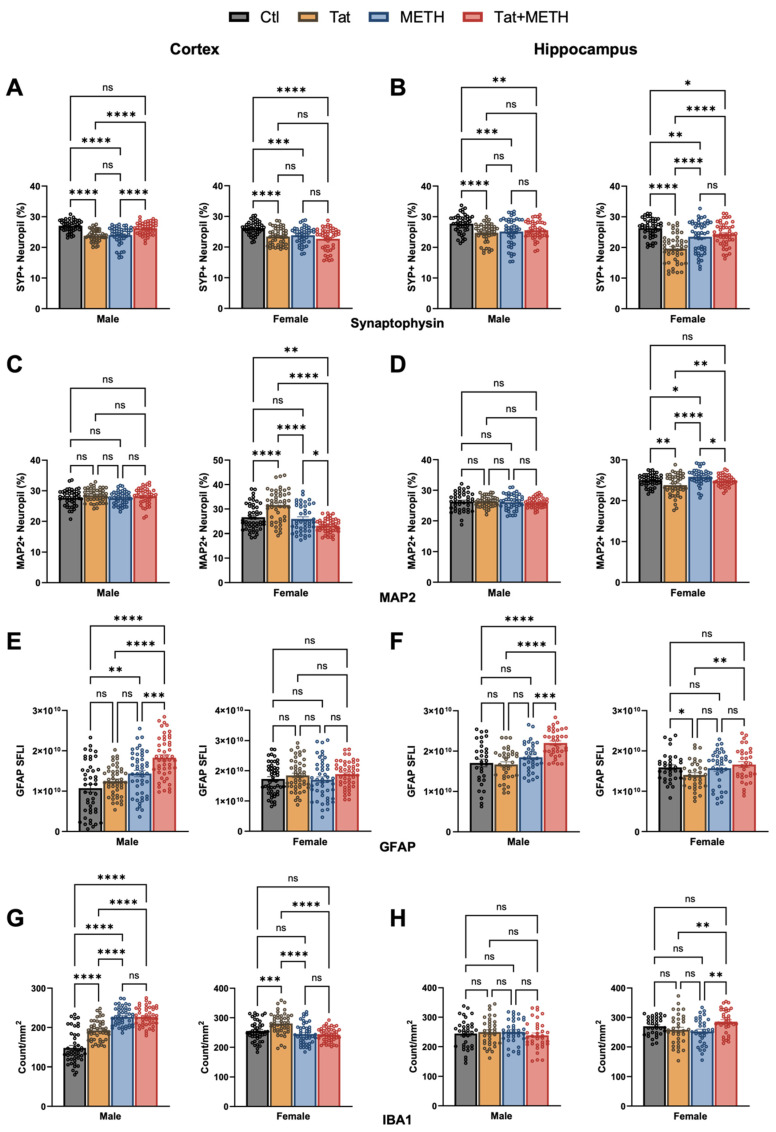
Quantitative results of histological analysis investigating neuronal injury, activation of astrocytes and microglia. (**A**) SYP^+^ neuropil percentage in cortex (LIII). (**B**) SYP^+^ neuropil percentage in hippocampus (CA1). (**C**) MAP2^+^ neuropil percentage in cortex (LIII). (**D**) MAP2^+^ neuropil percentage in hippocampus (CA1). (**E**) GFAP fluorescence in cortex (total). (**F**) GFAP fluorescence in hippocampus (CA1). (**G**) Count of Iba1^+^ cells normalized to area in cortex (total). (**H**) Count of Iba1^+^ cells normalized to area in hippocampus (CA1). Values are mean ± SEM, n = 4 males and 4 females per group (12 microscopic fields per animal), * *p* ≤ 0.05, ** *p* ≤ 0.01, *** *p* ≤ 0.001, **** *p* ≤ 0.0001; ANOVA and Fisher’s PLSD post hoc test. Details of the statistical analysis are presented in Supplementary Table S2.

Histological analysis was performed on brain tissues harvested from a randomly selected subset of the behaviorally tested mice as described in Materials and Methods (Figure 3A–H and Figure S2 include DAPI staining of nuclear DNA for panels A–D). Details of the statistical analysis are presented in Supplementary Table S2. Using deconvolution microscopy, we quantified the percentage of SYP and MAP2 positive neuropil as a measure of injury to pre-synaptic terminals and neurites, respectively. We analyzed non-overlapping fields of LIII of the cortex and the CA1 of the hippocampus since injury to these regions is linked to impairment of spatial and recognition memory, respectively. In the cortex of males, both Tat and METH alone significantly reduce the percentage of SYP^+^ neuropil (Figure 3A,B). However, in the presence of both Tat and METH, (Tat + METH), the percentage of SYP^+^ neuropil is similar to that of Ctl mice. This effect was not seen in the cortex of the female Tat + METH animals, where it remained reduced to the same extent as in the Tat and METH groups (Figure 4A). In the hippocampus, both male and female Tat animals showed a decrease of SYP^+^ neuropil compared to the respective Ctl animals (Figure 4B). In male animals, this reduction was similar in METH and Tat + METH groups. However, in the female hippocampus, the percentage of SYP^+^ neuropil is reduced in the METH and Tat + METH animals compared to Ctl, but still significantly higher than in the Tat group (Figure 4B).

Analysis of MAP2^+^ neuropil did not indicate any injury of neurites in male cortex or hippocampus (Figure 4C,D). Female animals, however, unexpectedly showed an increased MAP2^+^ neuropil in the cortex of Tat animals compared to Ctl, METH, and Tat + METH. That finding could reflect a disturbance of MAP2 homeostasis rather than the loss seen in hippocampus (Figure 4D). Females of the METH group had a MAP2^+^ neuropil equivalent to Ctl, whereas Tat + METH animals showed a decrease of MAP2^+^ neuropil to lower than the other three groups (Figure 4C). Analysis of MAP2^+^ neuropil in cortex and hippocampus indicated that neurites in the female brain were much more affected than in the male counterpart (Figure 4C,D). Altogether, exposure to Tat and METH, each alone and in combination, resulted in significant loss of presynaptic terminals, with the exception of male cortex, while Tat caused disparate disturbances of MAP2^+^ neurites only in females. Thus, a part of the overall neuronal injury occurred in a sex-dependent fashion. Moreover, the combination of Tat + METH seemed to avoid the loss of presynaptic terminals in LIII of male cortex.

### 3.3. Activation of Astrocytes and Microglia Due to Tat Expression and METH Treatment

To quantify activation of astrocytes and microglia, we stained tissue sections for astrocytic GFAP and microglial Iba1. Using immunofluorescence microscopy, we quantified fluorescence of GFAP indicating astrocyte activation and we quantified Iba1^+^ cells indicating the number of microglia. Increased GFAP fluorescence signifies astrocytosis and increased Iba1^+^ counts indicate microgliosis. The analysis focused on cortex and the CA1 region of the hippocampus (Figure 3E–H and Figure 4E–H). In the cortex, male METH and Tat + METH animals had increased GFAP fluorescence compared to Ctl. Compared to Tat mice, Tat + METH animals also showed a significant increase in fluorescence. In female animals, no significant changes were observed in the cortex between the groups (Figure 4E). In the hippocampus, male Tat + METH animals showed increased GFAP fluorescence compared to all three other groups. In female animals, Tat mice had a significant decrease in GFAP fluorescence compared to Ctl animals, whereas the GFAP fluorescence in METH and Tat + METH animals remained equivalent to Ctl (Figure 4F).

In the cortex, male animals showed an increased Iba1^+^ cell count in Tat animals compared to control (Figure 4G). Both METH and Tat + METH animals had an equivalent increase in Iba1^+^ cell counts. Increased Iba1^+^ cell count in male METH and Tat + METH animals were higher than the increase observed with Tat alone. Notably, the baseline count for microglia in the Ctl group was lower in males than females. In female animals, the Iba1^+^ cell count in the cortex was increased only in the Tat group compared to Ctl. The METH and Tat + METH animals showed a decreased Iba1^+^ cell count compared to Tat alone (Figure 4G). In the hippocampus of males, there were no significant changes seen in Iba1^+^ cell counts between groups. However, in the female animals, Tat + METH mice showed an increase in Iba1^+^ cells compared to both Tat and METH groups (Figure 4H). Together, these observations show sex- and brain region-dependent effects of Tat, METH, and their combination on markers of glial activation.

### 3.4. Modulation of RNA Expression Associated with Tat and METH Exposure

**Figure 5:**
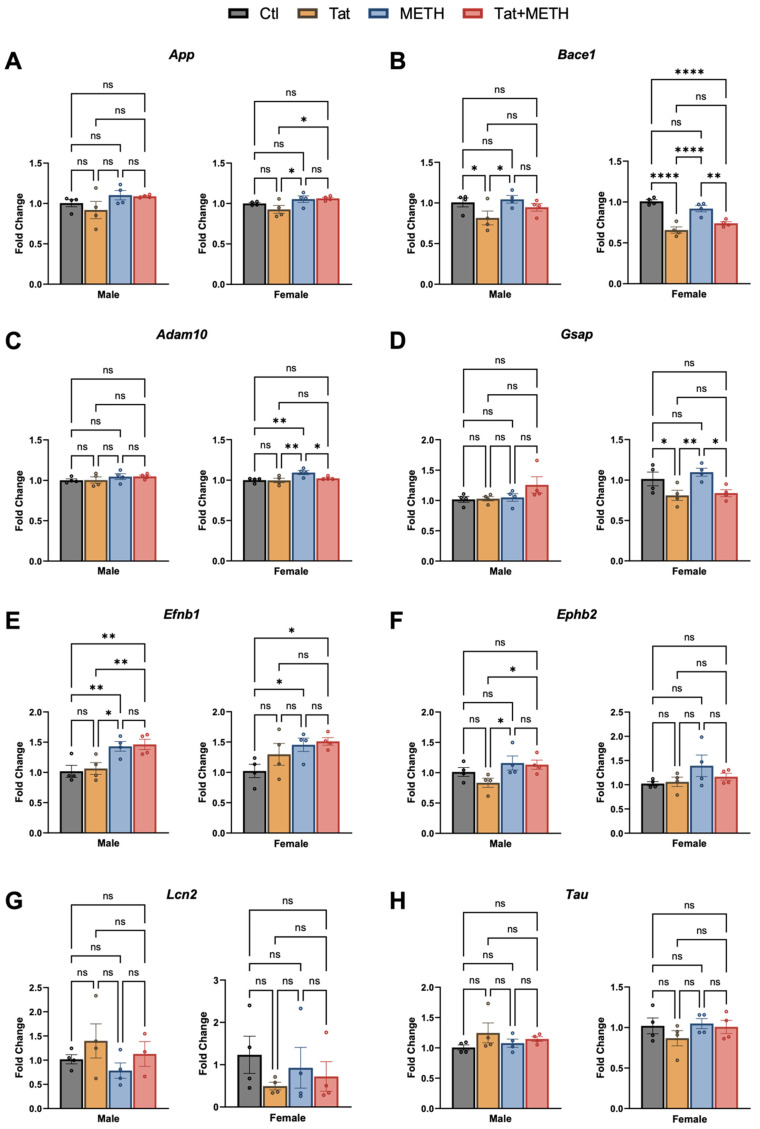
Changes in RNA expression of genes involved in AD, APP processing pathway, and inflammation are observed in cortex. (**A**) RNA analysis (qRT-PCR) of *App* expression showed an increased expression in female METH and Tat + METH animals. (**B**) Sex-dependent expression of *Bace1* was observed. (**C**) Again, we observed a sex-dependent modulation of gene expression in *Adam10*, with an increased expression in METH and Tat + METH females. (**D**) Expression of *Gsap* is decreased in Tat and Tat + METH females. (**E**) *Efnb1* expression is increased overall in METH and Tat + METH animals. (**F**) Expression of *Ephb2* is increase in male METH and Tat + METH animals compared to Ctl. (**G**) *Lcn2* expression is unchanged. (**H**) *Tau* expression is also unchanged. Values are mean ± SEM, *n* = 4 males and 4 females per group, * *p* ≤ 0.05, ** *p* ≤ 0.01, *** *p* ≤ 0.001, **** *p* ≤ 0.0001; ANOVA and Fisher’s PLSD post hoc test. Details of statistical analysis are presented in Supplementary Table S3.

RNA isolated from the cortex was analyzed by qRT-PCR to assess expression of genes involved in neurodegenerative diseases, such as Alzheimer’s disease (AD), since transgenic mouse models of AD show similarly loss of SYP^+^ neuropil [34]. Here we analyzed mRNA levels for amyloid precursor protein (*App*), *Tau*, and genes involved in the amyloid precursor protein (APP) processing pathways, β-amyloid converting enzyme-1 (*Bace1*), a disintegrin and metalloproteinase domain 10 (*Adam10*) and γ-secretase activating protein (*Gsap*) (Figure 5A–D,H). In addition, we assessed ephrin-B1 (*Efnb1*), ephrin receptor B2 (*Ephb2*), and lipocalin-2 (*Lcn2*) which are implicated in inflammation (Figure 5E-G)). Results are shown separately for male and female animals in Figure 5, but combined analysis and differences between female and male expression (normalized to Ctl male) were also analyzed (Figure S3). In female cortex, expression of *App* was higher in the METH compared to the Tat-only group and again in the Tat + METH animals, but no significant changes were observed in males (Figure 5A). *Bace1* expression was decreased in males of the Tat group compared to Ctl and METH animals (Figure 5B). The females showed a similar pattern in changes of *Bace1* expression but more robust, with Tat and Tat + METH dropping expression to levels lower than in Ctl or METH animals (Figure 5B). Expression of *Adam10* was not changed in male animals. However, in female animals, *Adam10* expression was higher in the METH animals compared to all three other groups (Figure 5C). The mean fold-change (FC) of expression of *Adam10* in the female Tat + METH group compared to Ctl is closer to the increase seen in the female METH than the Tat-alone group. Male animals showed no significant changes in expression of *Gsap* (Figure 5D), whereas in females, Tat and Tat + METH animals showed similar and lower expression of *Gsap* compared to Ctl and METH groups (Figure 5D). Expression of *Efnb1* in males of the Tat + METH group reached the same level as in METH animals, higher than in Ctl or the Tat group. A similar pattern was seen in female animals (Figure 5E). *Ephb2* expression was increased in males in METH and Tat + METH groups compared to the Tat animals, but largely unchanged in females (Figure 5F). The variability in *Lcn2* expression is seen in both male and female animals. Though the changes were n.s., the pattern of expression is different in animals expressing Tat and those exposed to METH (Figure 5G). The expression of *Tau* was largely unchanged, though males and females showed a different pattern between groups (Figure 5H). Overall, Tat and METH exposure seem to have a limited effect on the here tested genes, except for *Bace1* and *Efnb1* in both sexes and *Gsap* in females.

### 3.5. Analysis of RNA-Sequencing Data Reveals That Gene Modulation Associated with Tat Expression and METH Treatment Is Sex-Dependent

**Figure 6:**
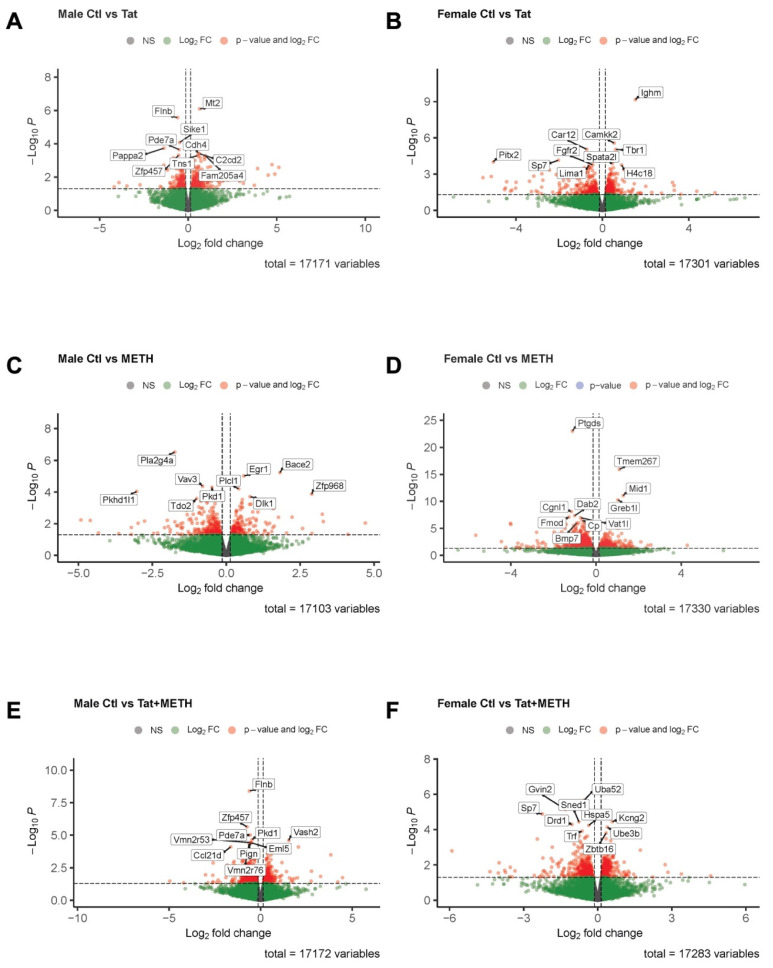
Changes in gene expression of hippocampus analyzed by RNA-Seq, reporting top 10 differentially regulated genes based on *p*-value. (**A**) Gene expression in male Tat compared to male Ctl. (**B**) Gene expression in female Tat compared to female Ctl. (**C**) Gene expression in male METH compared to male Ctl. (**D**) Gene expression in female METH compared to female Ctl. (**E**) Gene expression in male Tat + METH compared to male Ctl. (**F**) Gene expression in female Tat + METH compared to female Ctl. *n* = 3 males and 3 females per group, analyzed by DESeq2 (*p* ≤ 0.05).

**Figure 7:**
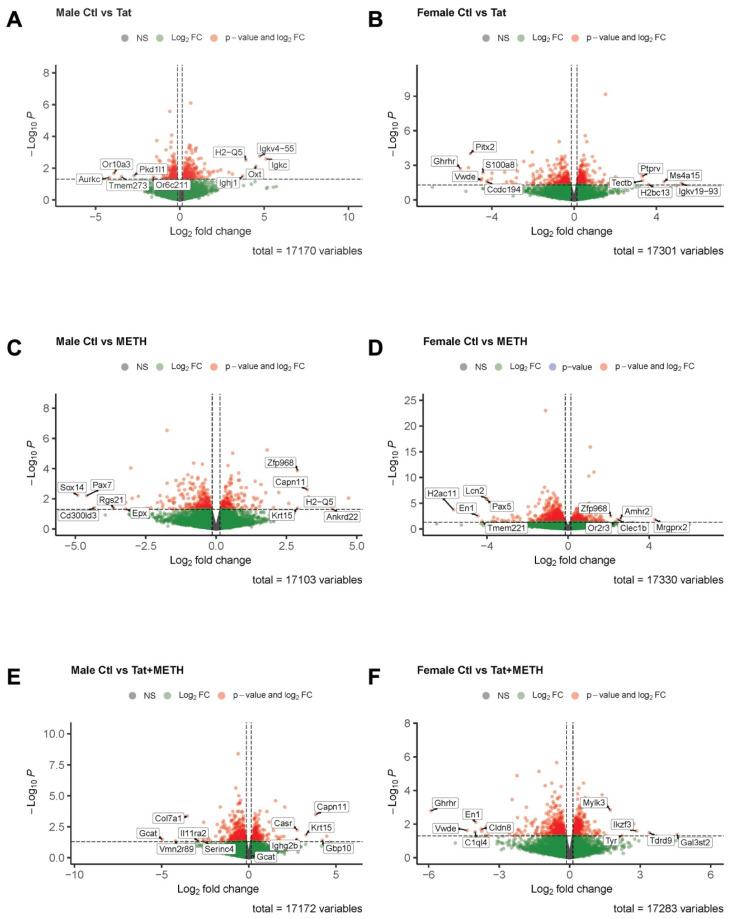
Changes in gene expression of the hippocampus analyzed by RNA-Seq, showing top 10 genes based on highest Log_2_ FC. (**A**) Gene expression in male Tat compared to male Ctl. (**B**) Gene expression in female Tat compared to female Ctl. (**C**) Gene expression in male METH compared to male Ctl. (**D**) Gene expression in female METH compared to female Ctl. (**E**) Gene expression in male Tat + METH compared to male Ctl. (**F**) Gene expression in female Tat + METH compared to female Ctl. *n* = 3 males and 3 females per group, analyzed by DESeq2 (*p* ≤ 0.05).

Since the qRT-PCR analysis of cortical mRNA revealed only relatively minor changes, we turned our attention to the hippocampus. However, the RNA quantity isolated from the hippocampus was more limited than that from cortex. Therefore, we decided for this study to utilize hippocampal mRNA for bulk RNA-sequencing (RNA-seq) in order to assess modulation of gene expression by Tat, METH, and Tat + METH. The data for the experimental groups were analyzed first separately by male/female groups and gene expression compared to the respective sex-specific Ctl groups (Figure 6 and Figure 7, Tables S1 and S2). In addition, combined group analysis was also performed to distinguish the sex-independent from sex-dependent effects in gene expression (Figures S4 and S5, Table S6).

First, we investigated differentially expressed genes based on *p*-value for change compared to Ctl animals (Figure 6, Table S4). In male Tat animals (compared to male Ctl) modulated genes are those involved in cAMP metabolic process, control of adhesion and autophagy, and upstream regulator of inflammation (Figure 6A [*Mt2*, *Flnb*, *Sike1*, *Pappa2*, *Pde7a*, *Cdh4*, *C2cd2*, *Zfp457*, *Tns1*, *Fam205a4*], Table S4). In male METH animals compared to male Ctl modulated genes are involved in lipid and progesterone metabolic processes, calcium homeostasis, APP processing, and regulation of immune response (Figure 6C [*Pla2g4a*, *Bace2*, *Egr1*, *Vav3*, *Pkd1*, *Plcl1*, *Pkhd1l1*, *Zfp968*, *Dlk1*, *Tdo2*], Table S4). In male Tat + METH animals (compared to male Ctl) modulated genes are involved in angiogenesis and vascular repair, cAMP metabolic processes, and calcium homeostasis (Figure 6E [*Flnb*, *Zfp457*, *Pde7a*, *Pkd1*, *Vash2*, *Vmn2r53*, *Pign*, *Eml5*, *Vmn2r76*, *Ccl21d*], Table S4). On the other hand, in female Tat animals compared to female Ctl modulated genes are involved in cell proliferation and migration, inflammation, and mitogen-activated protein kinase (MAPK) signaling pathway, and regulation of prolactin (Figure 6B [*Ighm*, *Camkk2*, *Car12*, *Tbr1*, *Sp7*, *Pitx2*, *Spata21*, *Fgfr2*, *H4c18*, *Lima1*], Table S4). In female METH animals (compared to female Ctl) modulated genes are involved in apoptosis, cell proliferation, iron transport and homeostasis, and the TGFβ signaling pathway (Figure 6D [*Ptgds*, *Tmem267*, *Mid1*, *Greb1l*, *Cgnl1*, *Dab2*, *Fmod*, *Vat1l*, *Cp*, *Bmp7*], Table S4). Finally, in female Tat + METH animals compared to female Ctl) modulated genes are involved in apoptosis, ubiquitin mediated proteolysis, iron transport, and neuronal growth and proliferation (Figure 6F [*Uba52*, *Gvin2*, *Sp7*, *Kcng2*, *Sned1*, *Drd1*, *Hspa5*, *Ube3b*, *Trf*, *Abtb16*], Table S4). Thus, the *p*-values for differentially regulated genes point to sex-dependent differences in the hippocampus of all experimental groups.

Second, we prioritized differentially expressed genes based on highest log-FC (*p* < 0.5, top five negative log-FC and top five positive log-FC) (Figure 7, Table S5). In male Tat animals compared to male Ctl downregulated genes are involved in mitosis and calcium homeostasis, whereas upregulated genes are involved in the oxytocin pathway and antigen presentation in the immune response (Figure 7A [*Aurkc*, *Or10a3*, *Tmem273*, *Pkd1l1*, *Or6c211*, *Igkc*, *Igkv4-55*, *Oxt*, *H2-Q5*, *Ighj1*], Table S5). In male METH animals compared to male Ctl) downregulated genes are involved in ERK signaling pathway, positive regulation of inflammation and eosinophil granulocytes, whereas upregulated genes are involved in arachidonic acid synthesis, and calcium-dependent signal transduction (Figure 7C [*Sox14*, *Pax7*, *Cd300ld3*, *Rgs21*, *Epx*, *Ankrd22*, *H2-Q5*, *Capn11*, *Krt15*, *Zfp968*], Table S5).

In male Tat + METH animals compared to male Ctl downregulated genes are involved in lipid metabolism and phospholipid biosynthetic process, whereas upregulated genes are involved in calcium-dependent signal transduction, calcium homeostasis, and antigen presentation (Figure 7E [*Gcat*, *Vmn2r89*, *Col7a1*, *Il11ra2*, *Serinc4*, *Gbp10*, *Capn11*, *Krt15*, *Casr*, *Ighg2b*], Table S5). On the other hand, in female Tat animals, compared to female Ctl, downregulated genes are involved in growth hormone synthesis, production of prolactin, and MAPK signaling pathways, whereas upregulated genes are involved in antigen presentation, ferroptosis inhibition, and cell proliferation (Figure 7B [*Ghrhr*, *Pitx2*, *Vwde*, *S100a8*, *Ccdc194*, *Igkv19-93*, *Ms4a15*, *H2bc13*, *Tectb*, *Ptprv*], Table S5). In female METH animals compared to female Ctl downregulated genes are involved in cell adhesion and migration, apoptosis and inflammatory response, whereas upregulated genes are involved in mast cell degranulation and migration (Figure 7D [*H2ac11*, *En1*, *Tmem221*, *Lcn2*, *Pax5*, *Mrgprx2*, *Amhr2*, *Clec1b*, *Or2r3*, *Zfp968*], Table S5). Finally, in female Tat + METH animals compared to female Ctl downregulated genes are involved in growth hormone synthesis and ERK signaling pathway, whereas upregulated genes are involved in the adaptive immune response (Figure 7F [*Ghrhr*, *Vwde*, *C1ql4*, *En1*, *Cldn8*, *Gal3st2*, *Tdrd9*, *Ikzf3*, *Tyr*, *Mylk3*], Table S5). Overall, whether assessed based on *p*-value or log-FC, the top 10 modulated genes in hippocampus are distinct between sexes and experimental groups.

Analysis of the data for females and males combined revealed as the top 10 differentially regulated genes based on *p*-value were *Igkc* and *Gnb3* in the Tat group, *Zfp968*, *Gnb3*, and *Pax7* in the METH group, but none in the Tat + METH group. However, the top 10 differentially expressed genes based on FC were *En1*, *Or10ac1*, and *Igkc* for the Tat group, *Pax7*, *En1*, and *Zfp968* for the METH group, and *Or10ac1* and *En1* for the Tat + METH group. Notably, *Zfp968* and *Pax7* are all regulators of RNA polymerase II, but *Sp7* is significantly differentially regulated only in females, and *Pax7* is detected as significantly changed only in the combined analysis in the METH group. *Zfp457* is another regulator of RNA polymerase II but only significantly differentially regulated in males in the Tat and Tat + METH groups. Thus, Tat and METH and their combination all significantly affect the regulation of RNA polymerase II but through different regulators, of which *Zfp457* and *Sp7* are modulated in a sex-dependent fashion.

We also compared the gene expression of females directly to that of males within their own experimental or treatment groups (Figure S6). This analysis revealed a class of widely expressed and evolutionary conserved dosage-sensitive regulators as the most differentially regulated genes that include the neighboring ubiquitously transcribed tetratricopeptide repeat containing Y-linked (*Uty*), DEAD-box helicase 3 Y-linked (*Ddx3y*) and eukaryotic translation initiation factor 2, subunit 3 (*Eif2s3y*), and the lysine demethylase 5D (*Kdm5d*). We also observed that prolactin (Prl) is significantly increased in expression in females when Tat is present, in Tat and Tat + METH groups, but not by METH alone (Figure S6B,D).

### 3.6. Analysis of RNA-Sequencing Data Using IPA Confirmed Sex-Dependent and Sex-Independent Responses

The RNA-seq results were further investigated by Ingenuity Pathway Analysis (IPA) for pathway and network analysis. The Top 10 modulated genes from each group were cross matched with those found in the top networks identified by IPA (Table S7). Compared to the Ctls, mice of the Tat group expressed increased Metallothionein 2 (*Mt2*) in males and females combined but the difference was driven by the increase in males). *Mt2* is involved in neurological disease, inflammatory response and organismal injury and abnormalities. In addition, only in female Tat mice compared to female Ctl mice did we see an increase in T-box brain protein 1 (*Tbr1*) and decrease in gamma-aminobutyric acid type A receptor subunit alpha 2 (*Gabra2*). *Tbr1* is a transcriptional repressor involved in cortical development and *Gabra2* is involved with inhibitory neurotransmission across chemical synapses and affects chloride channel and GABA-A receptor activity.

In the METH group compared to Ctl, female mice were overall more impacted, with a larger range of genes modulated. Genes that were found to be regulated in combined analysis but where the change is driven by alteration in females include *Dab2*, *Cp*, *Lcn2*, *Bmp7*, and *Ahnak*. DAB adaptor protein 2 (*Dab2*) and bone morphogenetic protein 7 (*Bmp7*) are mainly involved in embryonic and tissue development, whereas *Ahnak*, is a nucleoprotein required for neuronal cell differentiation. On the other hand, Ceruloplasmin (*Cp*) and Lipocalin 2 (*Lcn2*) are both involved in iron transport and regulation. Genes that were identified as regulated in the combined analysis but where change is driven by the male samples was *Egr1*. Early growth response 1 (*Egr1*) plays a role in cellular growth and proliferation and is involved in AKT signaling. Genes that were detected as significantly regulated only in the combined male and female analysis were *Slc7a11*, *Ptpn6*, and *Pax5*, which are involved in cell-to-cell signaling and interaction and cell-mediated immune response. *Slc7a11* is part of the sodium-independent anionic amino acid transport system, Xc(-), with high specificity for cysteine and glutamate. Moreover, among the genes that were modulated by exposure to METH, *Lcn2* is involved in lipid metabolism, inflammation, iron transport and infections and *Ptpn6*, a protein tyrosine phosphatase, that is involved in adaptive and innate immunity and has been linked to Alzheimer’s Disease as risk factor. Sex-dependent genes that were only modulated in females include *Ptgds* and *Mgp*. Prostaglandin D2 synthase (*Ptgds*) is vital in CNS functions and has an anti-apoptotic role in oligodendrocytes, whereas matrix glia protein (*Mgp*) is involved in calcium ion binding.

In Tat + METH mice compared to Ctl, dopamine receptor D1 (*Drd1*) is decreased in males and females, indicating a large effect to the dopaminergic system. *Drd1* is vital in modulation of chemical synaptic transmission and regulates learning and memory.

Tumor necrosis factor member 10 (*Tnfsf10*) and endoplasmic reticulum aminopeptidase 1 (*Erap1*) were also decreased in both male and female Tat + METH mice, indicating a dampened inflammatory response and altered regulation of cell death and survival. This finding fits with the protected behavioral performance in the BM task, but not the compromised NOR. In addition, solute carrier family 38 (*Slc38a6*) was decreased only in female Tat + METH mice, which affects transmembrane transport of glutamine. Together, these data show that Tat and METH, each alone and in combination, modulate a wide range of biological functions. Modulation of genes related to neurotransmission, ion transport, cell signaling, lipid metabolism, development and inflammation occur in both sex-dependent and -independent ways.

### 3.7. IPA Analysis of RNA-Sequencing Data Discloses Top Scoring Networks, Highlighting the Role of Tyrosine Hydroxylase and Fos

**Figure 8:**
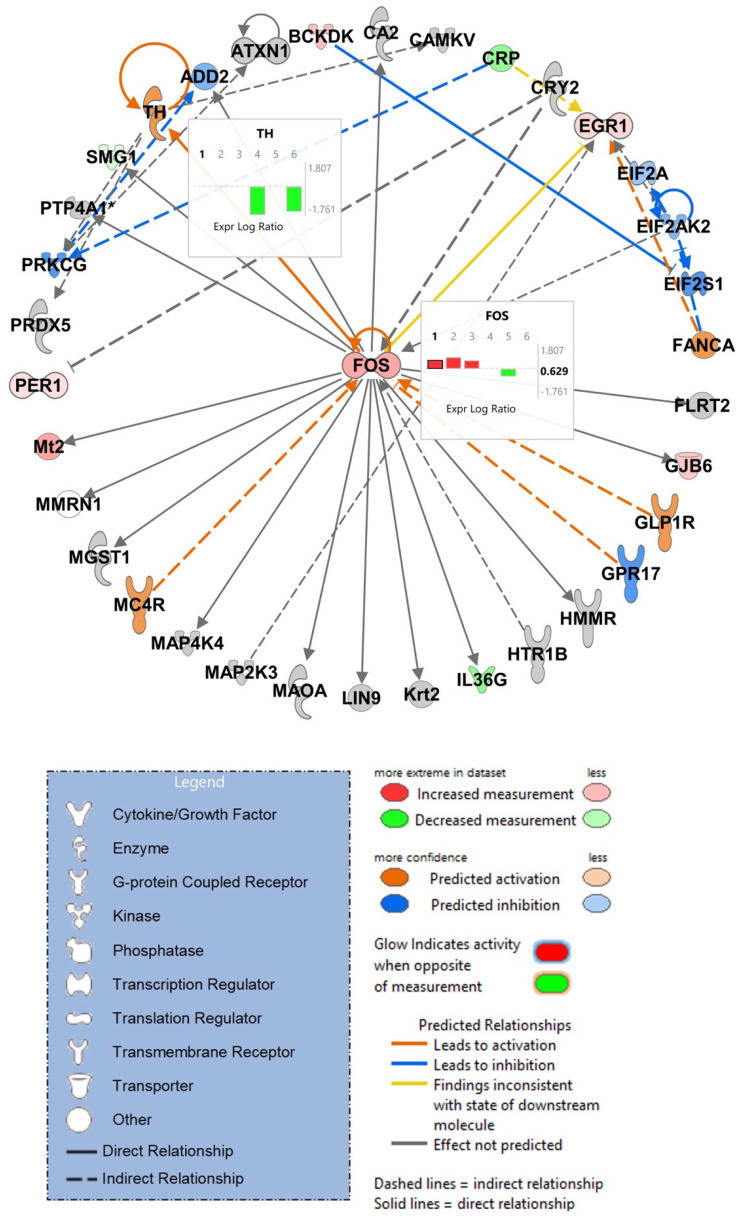
Ingenuity Pathway Analysis (IPA) software (version 2.2.1) generated network pathways with differential gene expression and predicted activation/inhibition by utilizing RNA-seq data of hippocampus. One of the top networks for male Ctl vs. Tat showed increased expression of *Fos* in groups 1–3, with decreased expression in group 5 (Insert FOS). This network also showed decreased expression of *Th* in group 4 and 6 (Insert TH). Thus, both *Fos* and *Th* display sex-dependent regulation in key components of the dopaminergic neurotransmission system. Groups: (1) Male Ctl vs. Tat, (2) Male Ctl vs. METH, (3) Male Ctl vs. Tat + METH, (4) Female Ctl vs. Tat, (5) Female Ctl vs. METH, and (6) Female Ctl vs. Tat + METH. (IPA analysis set at *p* < 0.01).

IPA was further used to predict networks which were significantly affected. One of the top networks observed indicated strong modulation of *Fos* as a key transcriptional regulator and tyrosine hydroxylase (*Th*) although these two genes were not among the 10 most significantly up- or downregulated genes as judged by *p*-value or FC (Figure 8). Expression of *Th* was reduced in female Tat and female Tat + METH animals compared to female Ctl. Expression of *Fos* was increased in male Tat, male METH, and male Tat + METH animals compared to male Ctl. On the other hand, expression of *Fos* was decreased for female METH animals compared to female Ctl. Overall, modulation of *Fos* is more affected in male animals, with increased expression, whereas expression of *Th* is highly affected in female animals expressing Tat.

## 4. Discussion

The use of drugs such as METH in PWH are well documented. METH use appears to increase the risk of NCI when associated with neuroHIV [1,3,7]. However, the mechanisms behind their combined effects are still not clearly understood. This study utilized a long-term, low-dose METH regimen in an in vivo model of neuroHIV based on inducible, transgenic expression of viral Tat, which revealed sexual dimorphism in learning and memory performance, brain injury and gene expression. The latter supports the notion of far-reaching effects of METH and Tat on neuroinflammation and neuronal injury that are in a large part sex and brain region specific.

Locomotion and optomotor tests in all experimental groups showed that the animals’ motor functions and vision were intact about 7 months after transient induction of viral Tat expression and 5 months after the last METH exposure. METH and Tat + METH promoted mobility in males with increased ambulation and center activity compared to animals in the Ctl and Tat groups. This is an interesting finding, as earlier studies investigating METH and locomotion found that females were more affected, showing increased locomotor activity compared to males [35,36,37]. Our finding supports the idea that sex-dependent effect may depend on the dosage of METH. In addition, the optomotor test also showed a METH dependent effect, where the number of head tracks were decreased in METH and Tat + METH groups compared to Ctl and Tat alone. Other studies have observed retinal neurodegeneration in animals receiving METH at 0.5 mg/kg for two months and also 6 mg/kg for five days, and our experimental protocol may be a case where the dosage has an effect on vision-driven responses [38,39].

In the BM task, no clear, significant sexual dimorphism was detected, but Tat + METH animals performed better compared to Tat- or METH-only groups. In the NOR test, however, females were more affected by Tat in two groups, Tat and Tat + METH. It is important to note that in the female control, the difference in time spent with the familiar and novel object did not reach the significant cut-off *p* ≤ 0.05 level (*p* = 0.0708). This may be associated with normal aging as diminishing performance in the NOR test has been shown to start as early as 10 months of age [40]. On the other hand, female animals receiving METH performed better in the NOR task compared to all other groups. This observation indicates that the areas of the brain that are responsible for performance in BM and NOR, respectively, may be differently affected by the presence of Tat and exposure to METH. BM is a behavioral task assessing spatial learning and memory, which mainly relies on hippocampal activity [41,42]. On the other hand, performance on the NOR test utilizes parts of the hippocampus (CA1/CA3), as well as other brain regions such as insular cortex, perirhinal cortex, and medial prefrontal cortex [43,44,45,46]. The effect that Tat and METH each independently and Tat + METH combined had on these specific locations in the brain may be distinct, thus leading to distinct outcomes of specific behavioral tests of memory performance.

The result of the histological analysis also supports sex- and region-dependent effects of Tat and METH, each alone and in combination. Analysis of synaptophysin was performed to detect injury to pre-synaptic terminals. In cortex of both, Tat and METH groups, males displayed lower SYP^+^ neuropil compared to Ctl or Tat + METH, indicating loss of pre-synaptic terminals. Interestingly, male Tat + METH had SYP^+^ neuropil comparable to Ctl. In contrast, in the male hippocampus, all three groups, Tat, METH, and Tat + METH had a lower SYP^+^ neuropil compared to control. A similar trend was seen in the female cortex, where all three groups of females, Tat, METH, and Tat + METH, showed similar amount of pre-synaptic injury compared to Ctl. The hippocampus of female animals showed a similar trend to female cortex, with the Tat group showing a larger loss of pre-synaptic terminals. Overall, compared to Ctl, other experimental groups predominantly showed some level of injury to pre-synaptic SYP^+^ terminals. Thus, while Tat and METH each alone cause lasting injury to presynaptic terminals in cortex and hippocampus, the male cortex seems to be an exception in the case of exposure to combined Tat + METH. This unexpected protective effect might contribute to the unimpaired performance of the Tat + METH group in the BM probe test. The underlying reason for the task- and brain region-specific protection remains to be elucidated. The fact that the protective effect does not extend to performance in the NOR task suggests that only specific neuronal circuitries can resist damage by Tat and METH.

Analysis of MAP2^+^ dendrites assessed the level of injury to postsynaptic neurites and indicated no significant effects in both cortex and hippocampus of males. In females, however, MAP2^+^ neuropil was most affected in the Tat-exposed compared to other groups, where it was increased in the cortex, but decreased in the hippocampus. This shows that the injury to neurites is Tat-driven, specifically in females, where the increased level in the cortex, may be the result of compensatory expression to counteract injury as previously seen in other studies or a disturbance of normal physiological turn-over resulting in accumulation of the protein [47].

Quantitative analysis of GFAP was performed to discern activation of astrocytes. In males, an increase seemed to be driven by METH exposure, in both the cortex and hippocampus. There was increased expression of GFAP suggesting activation of astrocytes in the METH and Tat + METH groups. In female mice, on the other hand, the cortex was not affected while only the hippocampus of Tat-expressing animals displayed a diminished expression of GFAP. This observation shows that the effect of METH on astrocyte activation differs between sexes.

Assessment of Iba1^+^ cell numbers served to estimate microglial activation. In the cortex of males, all three experimental groups displayed increased Iba1^+^ counts compared to Ctl. However, no such effect was seen in the male hippocampus, suggesting microglial activation differs by brain region. On the other hand, female Tat mice had increased Iba1^+^ cell counts compared to other groups in the cortex. Also, Tat + METH females showed increased Iba1^+^ cell counts compared to Tat and METH groups in the hippocampus, indicating another sex-dependent response of glial cells. Overall, the results observed in the histological analysis regarding neuroimmunomodulation and neuronal injury based on pre-synaptic loss and neurite health show that the effect observed may be specific to sex and brain region, as not only males and females show a different response, but the response between the cortex and hippocampus within each sex can also be distinct.

As both neuroHIV and use of METH are known to affect the risk of AD development, some genes involved in AD and in the amyloid precursor protein (APP) processing pathway were analyzed [48]. In addition, genes that we have found to be involved in inflammatory response regulation were also analyzed (*Efnb1*, *Ephb2*, and *Lcn2*) [29]. Similar to behavioral and histological findings, sexual dimorphism was seen in the differential expression of these genes in the cortex. As *Bace1* and *Gsap* are involved in the amyloidogenic part of the APP processing pathway, our results would support a conclusion that Tat expression possibly decreases production of Ab_42_ (amyloidogenic amyloid-beta) [49,50]. However, as this is an analysis of RNA expression, it is also possible that the mRNA levels are not fully reflective of the amount of functional beta- and gamma-secretases. *Adam10* expression was the highest in METH-exposed females, suggesting the possibility that more Ab_40_ (non-amyloidogenic amyloid-beta) is being produced after exposure to the psychostimulant. Follow-up studies will need to analyze the ratio of Ab_42_ vs. Ab_40_ peptides in order to clarify if the net result can potentially promote or slow a neurodegenerative, AD-like process in a sex dependent fashion [12,13]. In any case, our findings indicate that expression of Tat and exposure to METH affect the APP processing pathway.

In addition, we found that the expression of *Efnb1* and *Ephb2* are increased due to exposure to METH. *Efnb1* and *Ephb2* have been shown to promote neuroinflammation with increased expression during chronic inflammation [51]. Our observation fits with the notion that METH promotes components of a pro-inflammatory mechanisms and together with the histological findings suggests that these genes are involved in activation of astrocytes and microglia in males.

Sexual dimorphism was continuously seen throughout the results observed in the behavioral assessments, histological analysis, and gene expression. Using the hippocampal RNA-seq data, we were able to parse out differences in gene expression within and between male and female animal groups. Overall, we observed genes involved in pathways that were changed in all groups, such as those involved in cAMP metabolism, inflammation regulation, immune response, and calcium homeostasis. However, genes involved in iron transport and homeostasis, were distinctly modulated in female animals. Compared to female Ctl, female METH animals showed downregulation of *Dab2*, Lrp2/Lcn2 receptor, *Cp*, all factors important in iron transport, and downregulation of *Trf*, an essential iron binding and transport protein. Female Tat + METH animals also displayed downregulation of *Lcn2*, a chelator and iron binding protein. Iron accumulation has previously been shown after METH administration with levels similar to those observed during natural aging, and that creates a risk of neurodegeneration by way of oxidative stress [52,53]. Our data support the notion that exposure to METH may lead to iron accumulation by inhibition of cellular iron control mechanisms, and this mechanism may also display sexual dimorphism.

In addition, we also compared the gene expression of females directly to that of males within their own experimental group. This approach revealed a class of widely expressed and evolutionary conserved transcriptional regulators as the most differentially regulated genes. Those genes include the neighboring ubiquitously transcribed *Uty*, *Ddx3y*, and *Eif2s3y*, and the lysine demethylase *Kdm5d*. *Uty*, *Ddx3y*, and *Eif2s3y* are all Y chromosome-linked and *Kdm5d* activity is required for sex-dependent gene expression, which is in line with the significantly diminished expression in females independently of exposure to Tat, METH, or the combination thereof [54,55,56]. On the other hand, we observed that *Prl* is significantly increased in expression in females when Tat is present, in the Tat and Tat + METH groups. Change in the expression of *Prl* has been shown to affect cognition, memory/learning, stress, and anxiety [57]. *Prl* has also been shown to affect amyloid beta plaque load, Tau phosphorylation, microgliosis, and astrogliosis [58]. It plays a key role in neuroimmunomodulation, where treatment with *Prl* has been shown to cause a time- and dose-dependent increase in pro-inflammatory cytokines [59]. On the other hand, *Prl* has also been shown to have anti-inflammatory effects through its activation of STAT3 and IL-10 secretion [57]. In addition, *Prl* is known to modulate various downstream signaling pathways which include the Janus kinase/signal transducer and activator of transcription (JAK2/STAT5), MAPK extracellular regulated kinase 1/2 (ERK1/2), and phosphoinositide 3-kinase/protein kinase B (PI3K/AKT) [60,61]. In hippocampal neurons, these pathways are important in survival processes by regulating apoptosis [60]. Another study has shown that *Prl* treatment can affect expression of GABA receptors, which in turn can affect hippocampal parvalbumin (PV) expression and behavioral performance [62]. These previous studies have shown that *Prl* has the dual ability to be both neuroprotective and neurotoxic. Although the role that *Prl* plays in our animal model is uncertain, the increased expression especially in female Tat and Tat + METH animals may be a key to the observed sexual dimorphism. Furthermore, distribution of *Prl* receptors varies throughout the brain and thus may affect the different response seen in cortex versus hippocampus. *Prl* receptors may also differ in the brain regions responsible for behavioral performance on BM vs. NOR tests [59,63].

Moreover, using the top scoring networks predicted by IPA software, analysis of our hippocampal RNA-seq data showed a drop in *Th* expression in female Tat and Tat + METH groups. Inhibition of *Th* expression by treatment with Tat has been observed before in vitro and in vivo [64,65]. Expression of *Th* is essential as it is the rate limiting step in the biosynthesis of catecholamines, including dopamine, norepinephrine, and epinephrine [66]. However, this could also be the reason that in certain cases, the response in the Tat + METH group is improved compared to those animals receiving Tat or METH independently. Exposure to METH is known to increase the level of dopamine in the brain above baseline [67]. When this is combined with lowered expression of *Th* due to Tat, the two extremes may result in a less injurious level of dopamine closer to homeostasis. Additionally, the same network identified by IPA also displayed changes in *Fos* mRNA. Expression of *Fos* is increased in males in all three experimental groups compared to Ctl. Yet, in females, the METH group had a drop in *Fos* expression compared to Ctl. *Fos* is a marker for neuronal activation, and mice with a loss of c-*Fos* gene expression are unable to develop locomotor sensitization from psychostimulant drugs [68,69]. Increased expression of *Fos* in males could contribute to locomotor sensitization and a long-term increase in locomotor activity that was seen in males. However, if increased *Fos* expression also confers protection of spatial memory and cortical neurites in the Tat + METH condition remains to be elucidated. In females, IPA also noticed decreased expression of *Gabra2* in the Tat group compared to female Ctl. A knockdown of *Gabra2* generally causes learning and memory deficits but spares spatial learning and memory [70,71]. In addition, this decreased expression of GABA receptor was in line with the increased expression or *Prl* and the resulting hippocampal and behavioral disturbance [62]. The combined effects of modulating *Prl* and GABA receptor may explain the compromised performance of the female Tat mice in the NOR task.

Generally, both Tat and METH independently disturbed spatial memory, as seen in the BM. On the other hand, the combination of Tat + METH gradually ameliorated the compromising effect on spatial memory. In the NOR test, Tat and METH independently and in combination equally diminished recognition memory in males, but in females, only those exposed to Tat were compromised, not METH alone. These results regarding neuronal health and neuroinflammation reflect sex-dependent and brain region-specific responses. In addition, it is important to note that responses to METH and Tat exposure were lasting, as all animals received their final injection of METH at 7 months-of-age and behavioral assessments and tissue collection occurred at 12–13 months-of-age. Therefore, the responses might have been different if animals were analyzed immediately after the last METH injection. Overall, neuronal injury and neuroinflammation outweighed some potentially beneficial effects, suggesting the dosage of METH used in this study may still be too high to be neuroprotective. Desoxyn is the METH-based stimulant medication approved by the FDA for ADHD treatment. The recommended range for this drug, a methamphetamine hydrochloride tablet, is 20 to 25 mg daily [72]. In children averaging to be 50 lbs or 23 kg, this dosage would be lower than what was used here. It is possible that METH at the level used for ADHD therapy also benefits HAND patients. Long-term effects in HAND and ADHD patients, however, would still be to be carefully assessed, as limited information is available about Desoxyn users. Future studies will need to clarify if there is an optimal dose that can result in a therapeutic benefit for HAND, as well as ADHS that outweighs injurious effects and addiction-inducing risks.

The differences in the effects of Tat and METH on the outcomes of two memory tasks, on histological measures of brain injury, on gene expression, on different brain regions and sexes all may appear as being inconsistent, but that is in line with other studies. One earlier study employing the same iTat mouse model confirmed induction of the viral protein by Dox but detected no effect on spatial memory and reversal learning within 10 days of Tat induction [73]. Another study focusing on the impact of METH only found inconsistent effects on neurochemical parameters in three brain regions which also depended on the dosing regimen of the psychostimulant [74]. These two studies did not report any sex-dependent findings. One study investigating the effects of METH on iTat mice used only males and found that the combination of Tat + METH resulted in better visual discrimination learning than with Tat or METH alone, while compromising cognitive flexibility in reversal learning [75]. In fact, the authors found that Tat alone improved early but worsened late phase reversal learning, and concluded that Tat and METH affect specific domains of cognition with possible improvement under certain conditions. Another study with iTat mice induced expression with Dox for 12 weeks before a 5-week METH exposure [76]. This experimental protocol resulted in decreased working and spatial memory in iTat mice receiving METH. The study also found inconsistent effects of METH and Tat on synaptic markers across four brain regions but sex-dependent effects were not recorded. A study that investigated electrophysiological effects of HIV using iTat mice found an increase of spontaneous excitatory postsynaptic currents compared to Tat-negative controls in both sexes, but observed deficits on inhibitory control only in females [77]. Another study of electrophysiological alterations in iTat compared to Tat-negative control brains used the same induction protocol than this study, Dox for seven consecutive days, but employed only males. The authors observed opposing effects of Tat on neuronal excitation in cortex and hippocampus directly after induction of transgene expression [78]. Our study indicates that brain region-specific and sex-dependent, inconsistent effects of Tat and METH are long-lasting.

The limitations of this study are that it investigated the effect of only one viral factor and one METH regimen, and analysis of protein expression was limited to the histological markers of neuronal injury and glial activation. All other changes were investigated at mRNA level. However, this study lays the groundwork and provides the rationale for further investigations, including at the protein level by proteomic analysis.

## Figures and Tables

**Table 1:** Primer sequences (mouse) used in qRT-PCR experiments.

Primer Name	Primer Sequence (5′-3′)
*App*	(Fwd) GGC CCT CGA GAA TTA CAT CA (Rev) GTT CAT GCG CTC GTA GAT CA
*Adam10*	(Fwd) ATG GTG TTG CCG ACA GTG TTA (Rev) GTT TGG CAC GCT GGT GTT TTT
*Bace1*	(Fwd) ATG TGG AGA TGA CCG TAG GC (Rev) TAC ACA CCC TTT CGG AGG TC
*Gsap*	(Fwd) ATG GCT GCA CTG CAC TGT AT (Rev) AGC TCA GAC ACC ATC AAG CC
*Efnb1*	(Fwd) ACC CTA AGT TCC TAA GTG GGA (Rev) CTT GTA GTA CTC GTA GGG C
*Ephb2*	(Fwd) TAC ATC CCC CAT CAG GGT GG (Rev) GCC GGA TGA ATT TGG TCC GC
*Lcn2*	(Fwd) CCA GTT CGC CAT GGT ATT TT (Rev) GGT GGG GAC AGA GAA GAT GA
*Tau*	(Fwd) TGA GGG ACT AGG GCA GCT AA (Rev) CTG CCT TCC TCA CCT CTG TC
*Gapdh*	(Fwd) AGG TCG GTG TGA ACG GAT TTG (Rev) TGT AGA CCA TGT AGT TGA GGT CA

## Data Availability

The original data presented in the study are openly available in NCBI’s Gene Expression Omnibus and are accessible through GEO Series accession number GSE255016 (https://www.ncbi.nlm.nih.gov/geo/query/acc.cgi?acc=GSE255016).

## Supplementary Materials

The following supporting information can be downloaded at: https://www.mdpi.com/article/10.3390/v17030361/s1, Figure S1. Behavioral tests to assess vision and anxiety; Figure S2. Immunofluorescence staining of Synaptophysin and MAP2 in cortex and hippocampus with DAPI staining (imaged at 40×); Figure S3. Changes in RNA expression of genes involved in AD, APP processing pathway, and inflammation are observed in cortex; Figure S4. Volcano plots representing RNA-seq data derived from brain tissue from hippocampus, visualizing the top 10 differentially expressed genes based on *p*-value, sexes-combined analysis; Figure S5. Volcano plots representing RNA-seq data analyzing brain tissue from hippocampus, visualizing the top 10 genes modulated (cutoff *p*-value < 05) with largest log2 fold change; Figure S6. Volcano plots representing RNA-seq data analyzing brain tissue from hippocampus, visualizing top genes modulated in female animals compared to male animals; Table S1. Statistical analysis for data shown in Figure 2 (Excel file); Table S2. Statistical analysis for data presented in Figure 4 (Excel file); Table S3. Statistical analysis for data displayed in Figure 5 and Figure S3 (Excel file); Table S4. Biological functions and pathways for top 10 modulated genes based on *p*-value (Excel file); Table S5. Biological functions and pathways for top 10 modulated genes based on Log2 FC (Excel file); Table S6. Biological functions and pathways for top 10 modulated genes based on *p*-value in treated animals (combined) compared to control (combined) (Excel file); Table S7. Sex-dependent and sex-independent responses observed in Ingenuity Pathway Analysis (IPA) of hippocampal RNA-sequencing data (Excel file).

## Abbreviations

The following abbreviations are used in this manuscript:

cART	Combined antiretroviral therapy
PWH	People with HIV
NCI	Neurocognitive impairment
HAND	HIV-associated neurocognitive disorders
neuroHIV	Neuropathological features of HIV infection of the brain
HIVE	HIV encephalitis
METH	Methamphetamine
ADHD	Attention deficit hyperactivity disorder
Tat	HIV-1 Transactivator of transcription
BM	Barnes maze
NOR	Novel object recognition
APP	Amyloid precursor protein
TBI	Traumatic brain injury
AD	Alzheimer’s disease
Dox	Doxycycline
GFAP	Glial fibrillary acidic protein
Tet	Tetracycline
IACUC	Institutional animal care and use committees
TSRI	The Scripps Research Institute
LDT	Light/dark transfer
LM	Locomotor
OM	Optomotor
IF	Immunofluorescence
MAP2	Microtubule-associated protein 2
Iba1	Ionized calcium-binding adaptor molecule 1
LIII	Layer 3
CA1	Cornu ammonis 1
SYP	Synaptophysin
IPA	Ingenuity pathway analysis
Th	Tyrosine hydroxylase
