# Supplementary material for: Chronic, Low-Dose Methamphetamine Reveals Sexual Dimorphism of Memory Performance, Histopathology, and Gene Expression Affected by HIV-1 Tat Protein in a Transgenic Model of NeuroHIV
Source: Viruses. 2025 Feb 28;17(3):361. doi: 10.3390/v17030361 (PMC11946854; doi:10.3390/v17030361)
Supplement: Supplementary file 1 [file viruses-17-00361-s001.zip › viruses-3447226-Supplementary.pdf]

Chronic, Low-dose Methamphetamine Reveals Sexual Dimorphism in Memory Performance, Histopathology and Gene Expression Affected by HIV-1 Tat Protein in a Transgenic Model of NeuroHIV

Supplementary Materials

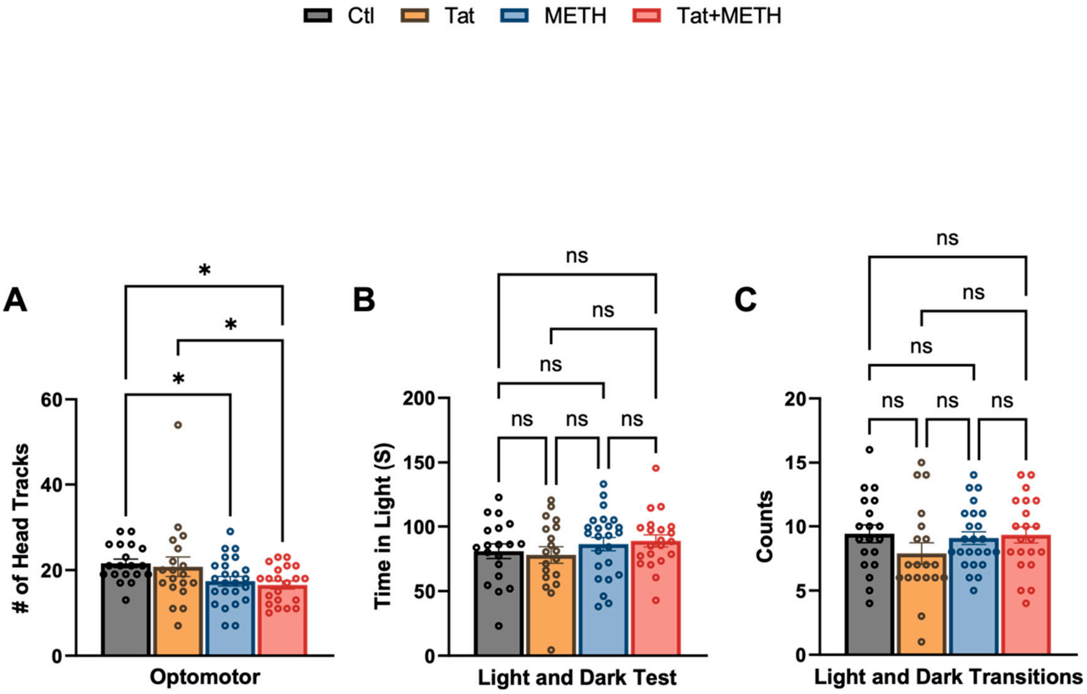

Figure S1. Behavioral tests to assess vision and anxiety. (A) Optomotor vision test shows visual ability of animals were intact, as all animals were able to track the moving stripes (all animals had counted # of head tracks). (B-C) Light/dark transfer test shows no difference between each group of animals, showing there were no observed anxiety-like behavior in the animals. Values are mean ± SEM, *p≤0.05, **p≤0.01, ***p≤0.001, ****p≤0.0001; ANOVA and Fisher's PLSD post hoc test. Details of the statistical analysis are presented in Supplementary Table S1

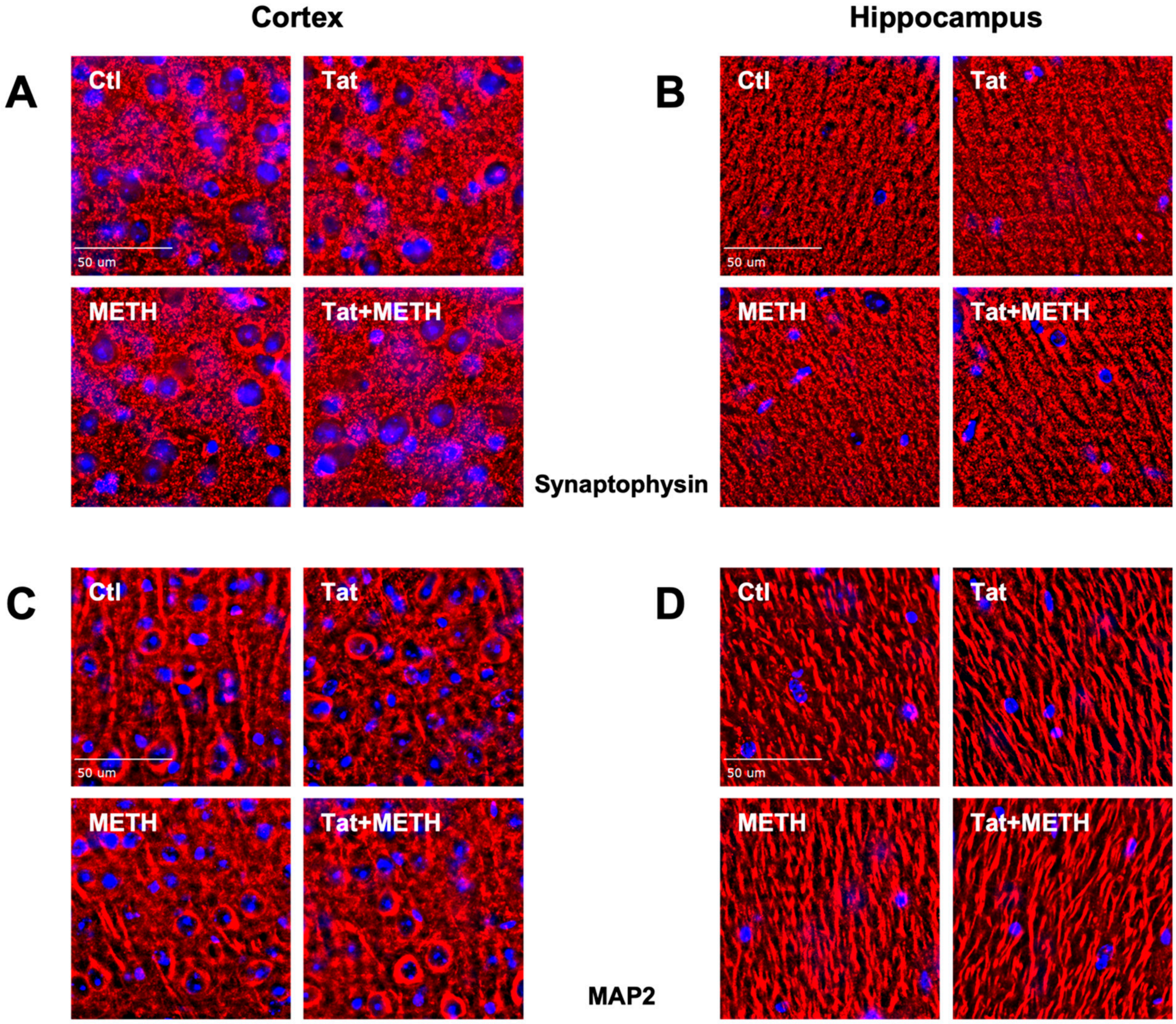

Figure S2. Immunofluorescence staining of Synaptophysin and MAP2 in the cortex and hippocampus with DAPI staining (imaged at 40x). Images of sagittal brain tissue sections stained for synaptophysin in the (A) LIII of the cortex and (B) CA1 of the hippocampus. Images of tissue stained for MAP2 in the (C) LIII of the cortex and (D) CA1 of the hippocampus. Representative images taken from male animals are shown. Scale bar = 50 μm

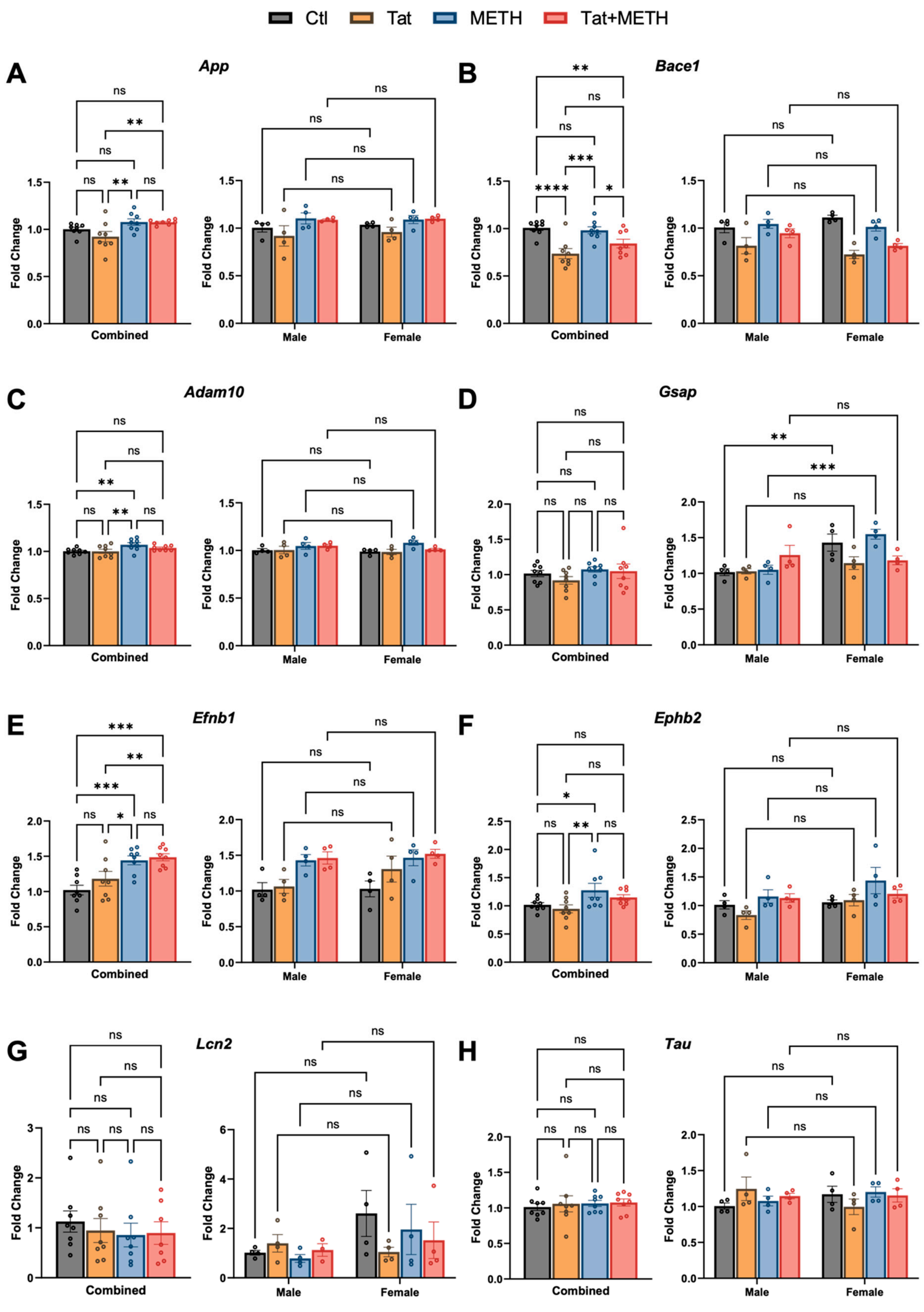
Ctl
Tat
METH
Tat+METH
A
App
B
Bace1
C
Adam10
D
Gsap
E
Efnb1
F
Ephb2
G
Lcn2
H
Tau
Fold Change
Combined
Male
Female
ns
*
**
***
****

Figure S3. Changes in RNA expression of genes involved in AD, APP processing pathway, and inflammation are observed in the cortex. (A) RNA analysis of APP expression showed an increased expression in METH and Tat+METH compared to Tat. (B) Expression of BACE1 is decreased in Tat and Tat+METH. (C) ADAM10 expression is higher in METH compared to Ctl and Tat. (D) Expression of GSAP is unchanged when combined, but there are sex differences in the Ctl and METH animals, with females expressing at a higher level. (E) EFNB1 expression is increased overall in METH and Tat+METH animals. (F) Expression of EPHB2 is increased in male METH compared to Ctl and Tat. (G) LCN2 expression is unchanged. (H) TAU expression is also unchanged. Values are mean ± SEM, n = 4 males and 4 females per group, *p≤0.05, **p≤0.01, ***p≤0.001, ****p≤0.0001; ANOVA and Fisher's PLSD post hoc test. Details of the statistical analysis are presented in Supplementary Table S3

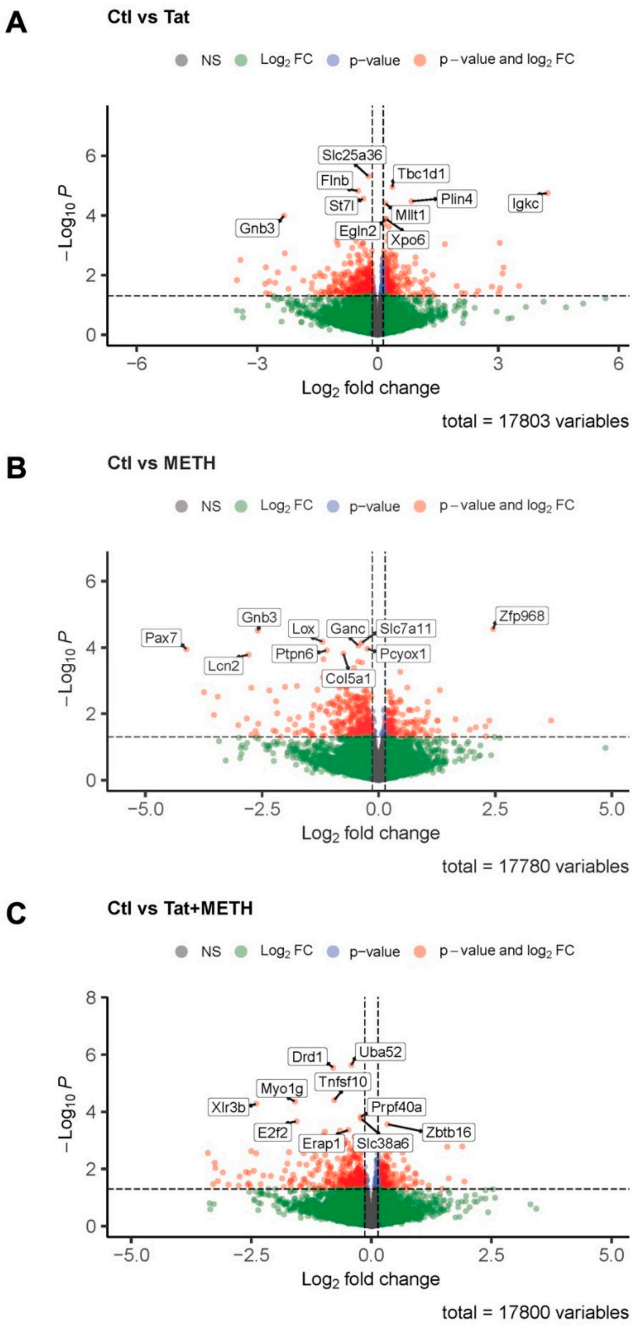

Figure S4. Volcano plots representing RNA-seq data analyzing brain tissue from the hippocampus, visualizing the top 10 differentially expressed genes based on p-value, sexes-combined analysis. (A) Genes that are modulated in all Tat animals compared to all Ctl animals based on the top p-value. (B) Genes that are modulated in all METH animals compared to all Ctl animals based on the top p-value. (C) Genes that are modulated in all Tat+METH animals compared to all Ctl animals based on the top p-value. The log2 fold changes indicate level of gene expression compared to Ctl (x-axis) and p-values are indicated as negative log(10) transformed (y-axis).

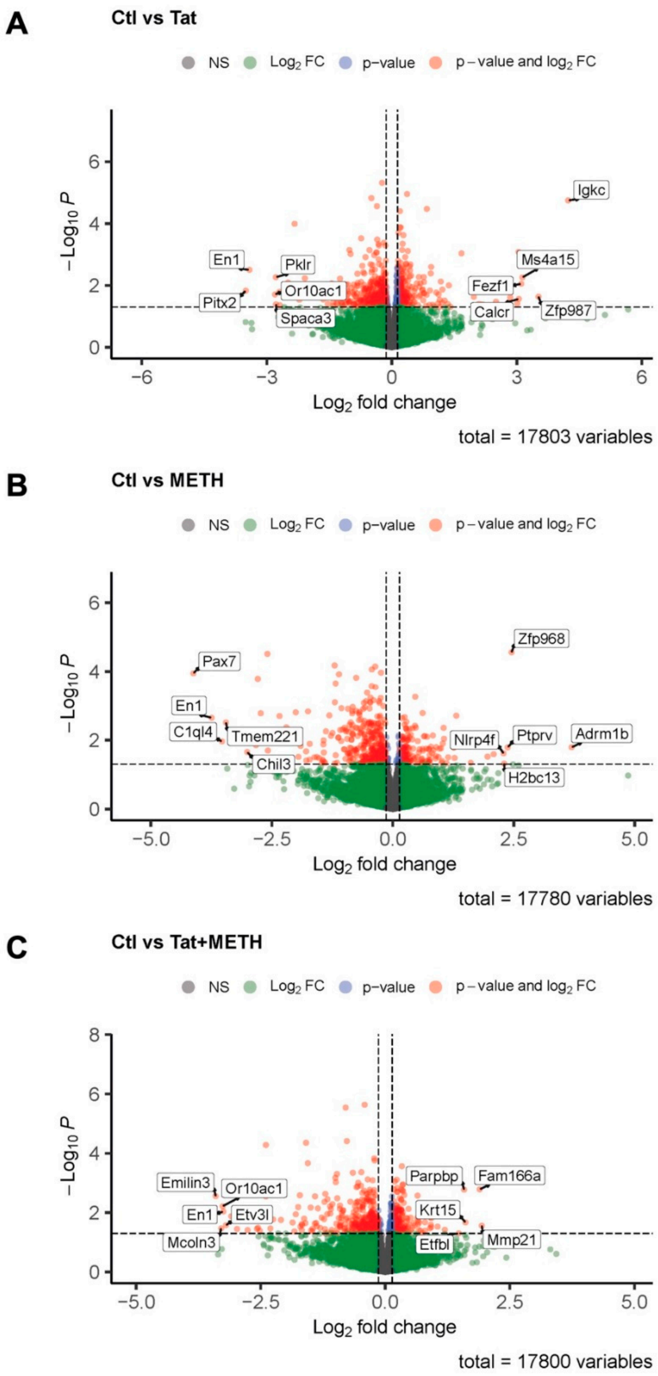

Figure S5. Volcano plots representing RNA-seq data analyzing brain tissue from the hippocampus, visualizing the top 10 genes modulated (cutoff p-value < .05) with the largest log2 fold change. (A) Genes that are modulated in all Tat animals compared to all Ctl animals based on the top log2 fold change value. (B) Genes that are modulated in all METH animals compared to all Ctl animals based on the top log2 fold change value. (C) Genes that are modulated in all Tat+METH animals compared to all Ctl animals based on the top log2 fold change value. The log2 fold changes indicate level of gene expression compared to Ctl (x-axis) and p-values are indicated as negative log(10) transformed (y-axis).

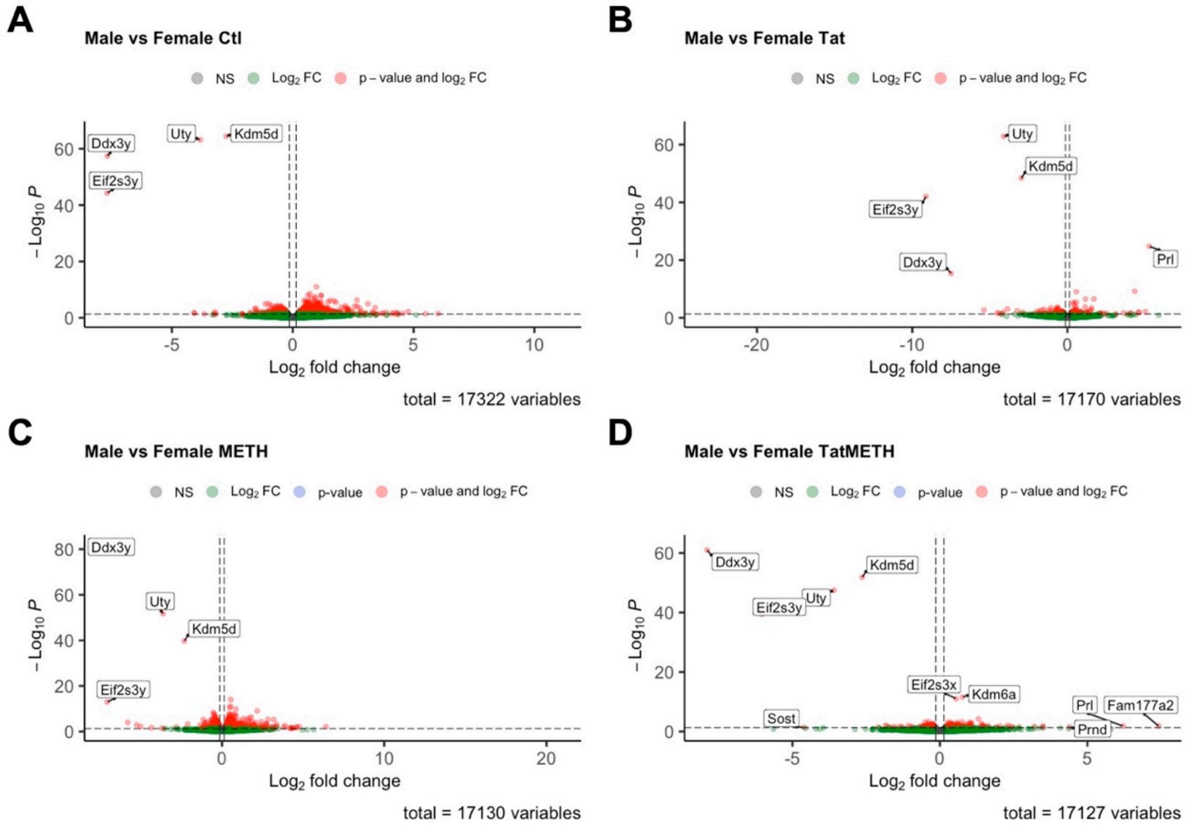

Figure S6. Volcano plots representing RNA-seq data analyzing brain tissue from the hippocampus, visualizing the top genes modulated in female animals compared to male animals. (A) Genes that are modulated in female Ctl animals compared to male Ctl animals. (B) Genes that are modulated in female Tat animals compared to male Tat animals. (C) Genes that are modulated in female METH animals compared to male METH animals. (D) Genes that are modulated in female Tat+METH animals compared to male Tat+METH animals. The log2 fold changes indicate level of gene expression compared to Ctl (x-axis) and p-values are indicated as negative log(10) transformed (y-axis).

Table S1. Biological functions and pathways for the top 10 modulated genes based on p-value. Genes represented in volcano plots shown in Figure 6. Highlighted genes are ones that were also observed in a different group, similar genes highlighted with corresponding color.

Table S2. Biological functions and pathways for the top 10 modulated genes based on Log_2 FC. Genes represented in volcano plots in Figure 7. Highlighted genes are ones that were also observed in a different group, similar genes highlighted with corresponding color.

Table S3. Biological functions and pathways for the top 10 modulated genes based on p-value in treated animals (combined) compared to control (combined). Genes represented in volcano plots in Figure S3-S4. Highlighted genes are ones that were also observed in a different group, similar genes highlighted with corresponding color.

Table S4. Sex-dependent and sex-independent responses observed in Ingenuity Pathway Analysis (IPA) of hippocampal RNA-sequencing data. Pathway and network analysis performed with IPA revealed top networks that overlap with those in which the top 10 modulated genes are involved. The table displays the modulation of top networks in each treated groups vs control, whether it is sex-dependent (sex specific treated group vs sex specific control) or sex-independent (combined treated group vs combined control). The diseases and functions to which the network is linked are reported together with the values of the log-FC, p-value and adjusted p-values. (*Notes 1 indicate the gene # observed in the top 10 modulated genes).

Table S5. Statistical analysis data for Figure 2. Statistical analysis raw data for results reported in the locomotor test (LM), Barnes maze (BM), and novel-object recognition test (NOR). These main behavioral findings are reported in Figure 2.

Table S6. Statistical analysis data for Figure 4. Statistical analysis raw data for results reported in the histological analysis of the cortex and hippocampus. Histological analysis was analyzed for synaptophysin, MAP2, GFAP, and IBA1. These histological findings are reported in Figure 4.

Table S7. Statistical analysis data for Figure 5. Statistical analysis raw data for results reported from qRT-PCR of cortex tissue. Changes in gene expression was analyzed for *App*, *Bace1*, *Adam10*, *Gsap*, *Efnb1*, *Ephb2*, *Lcn2*, and *Tau*. The result of gene expression modulation in the cortex are reported in Figure 5.
